# A Combined Epithelial Mesenchymal Transformation and DNA Repair Gene Panel in Colorectal Cancer With Prognostic and Therapeutic Implication

**DOI:** 10.3389/fonc.2020.595182

**Published:** 2021-01-15

**Authors:** Xiaoliang Huang, Jungang Liu, Haizhou Liu, Xianwei Mo, Yongsheng Meng, Lihua Zhang, Yuqing Deng, Yawei Zhang, Weizhong Tang

**Affiliations:** ^1^ Division of Colorectal & Anal Surgery, Department of Gastrointestinal Surgery, Guangxi Medical University Cancer Hospital, Nanning, China; ^2^ Guangxi Clinical Research Center for Colorectal Cancer, Nanning, China; ^3^ Department of Environmental Health Sciences, Yale School of Public Health, New Haven, CT, United States; ^4^ Department of Research, Guangxi Medical University Cancer Hospital, Nanning, China

**Keywords:** DNA repair, epithelial to mesenchymal transition, colorectal cancer, immunotherapy, metabolism

## Abstract

**Background:**

Epithelial mesenchymal transformation (EMT) and DNA repair status represent intrinsic features of colorectal cancer (CRC) and are associated with patient prognosis and treatment responsiveness. We sought to develop a combined EMT and DNA repair gene panel with potential application in patient classification and precise treatment.

**Methods:**

We comprehensively evaluated the EMT and DNA repair patterns of 1,652 CRC patients from four datasets. Unsupervised clustering was used for classification. The clinical features, genetic mutation, tumor mutation load, and chemotherapy as well as immunotherapy sensitivity among different clusters were systematically compared. The least absolute shrinkage and selection operator regression method was used to develop the risk model.

**Results:**

Three distinct CRC clusters were determined. Clustet1 was characterized by down-regulated DNA repair pathways but active epithelial markers and metabolism pathway and had intermediate prognosis. Clustet2 was characterized by down-regulated both epithelial markers and DNA repair pathways and had poor outcome. Clustet3 presented with activation of DNA repair pathway and epithelial markers had favorable prognosis. Clustet1 might benefit form chemotherapy and Clustet3 had a higher response rate to immunotherapy. An EMT and DNA repair risk model related to prognosis and treatment response was developed.

**Conclusions:**

This work developed and validated a combined EMT and DNA repair gene panel for CRC classification, which may be an effective tool for survival prediction and treatment guidance in CRC patients.

## Background

Colorectal cancer (CRC) remains a major cause of cancer-related mortality worldwide despite advancements in tumor screening, early diagnosis, and curative resection (1). Staging based on the tumor, nodule, and metastasis (TNM) is generally considered as the main tools for routine prognostication of CRC patients in treatment practice (2, 3). However, heterogeneity of clinical process and treatment response are often observed between individuals in the same stage, which are often attributed to diversity of CRC (4). The diversity of tumors is also manifested at the molecular level. Tumors of the same histological subtype may have different genetic backgrounds and gene expression profile. Tumors of different histological subtype may share common genetic backgrounds and molecular features. Identifying tumor subtypes with different molecular characteristics and clinical outcome is important for the precise treatment of cancer.

In recent years, the molecular classification of CRC has received increasing attention. The international CRC Subtyping Consortium developed a transcriptomic classification of colorectal cancer, which classifies CRC into four biologically distinct consensus molecular subtypes (CMSs). CMS1 and CMS4 tumors have high levels of immune infiltration but antagonistic functional orientation. CMS2 and CMS3 are devoid of immune cell infiltration (5). CMS4 subtype has the worst prognosis. The French national Cartes d’Identite´ des Tumeurs (CIT) program identified six molecular subtypes with distinct clinicopathological characteristics and molecular alterations (6). C1 (CIN_ImmuneDown_) is more frequently chromosomal instability (CIN) and immunosuppression. C2 (dMMR) contains most deficient mismatch repair (dMMR) tumors. C3 (KRASm) is enriched for KRAS-mutant tumors. C4 (CSC) is characterized by presenting cancer stem cell (CSC) phenotype–like gene expression profile as well as up-regulating of the bottom crypt signature. C5 (CIN_WntUp_) has frequency CNI with up-regulation of Wnt pathway. C6 is enriched for “normal-like’” tumor (7). Nevertheless, some defect limits the clinical application of the above-mentioned classification. There is no consensus on whether classification is associated with treatment response. Besides, tumor classification is based on whole-genome gene expression patterns, which increases the complexity of classification and decreases the feasibility of clinical application. And there is overlap between pathways enriched in different classification, increasing the uncertainty of the interpretation of the results. Selecting characteristic pathways for tumor classification may be a way to simplify the classification process and improve clinical utility, and assess the correlation between classification and treatment response.

Epithelial–mesenchymal transition (EMT) facilitates the acquisition of stem cell characteristics and sustains stem cell-like populations (8). During the process of EMT, cancer cells lose their epithelial morphology and adopt a spindle‐shaped and mesenchymal appearance progressively. Activation of EMT provides cancer cells with the enhanced plasticity required for invasion and metastasis (9). In CRC, EMT is strongly associated with tumor proliferation, infiltration, metastasis, tumor budding and drug resistance (10). Patients with active EMT tumor have poor prognosis. However, EMT is a reversible process, which offers new insight for the treatment of tumors (11). Incorporating EMT gene expression profiles into CRC classification may identify a subtype of cancer with high malignancy and therapeutic implications.

DNA repair is a critical system for recognizing and repairing abnormalities in the structure or sequence of DNA. Mutations in DNA repair genes, including mismatch repair (MMR), can impair cells’ ability to repair damaged DNA, leading to cell death or genome instability (12). Tumors with aberrant DNA repair pathway have increased mutational and neoantigen burden (13), which in turn were linked with greater tumor infiltration by activated T cells. DNA repair defects are associated with improved clinical response to PD-1 blockade, specifically, in CRC patients with deficient mismatch repair (dMMR) (14).

Therefore, we integrated EMT and DNA repair genes for CRC classification. Three CRC clusters with distinct prognosis and molecular characteristic were determined.

## Materials and Methods

### Clinical Specimens

In the present study, eight cases of CRC samples including two cased of metastatic CRC samples and six cased of non-metastatic CRC samples were obtained from patients at the Guangxi Medical University Cancer Hospital. The samples were subjected to RNA sequencing. All of the patients were pathologically diagnosed as CRC without chemotherapy or radiotherapy before the collection of the tissues. Written informed consents were obtained from all patients. The study was approved by the Ethics and Human Subject Committee of Guangxi Medical University Cancer Hospital. All experiments and methods were performed according to relevant guidelines and regulations formulated by the Guangxi Medical University.

### RNA-Seq Analysis

Total RNA was extracted using Trizol reagent (Invitrogen). The construction of RNA-seq library was based on the protocol of the IlluminaTruSeq RNA Sample Preparation Kit (illumina). Finally, RNA-seq analysis was performed by GENE+ company (Beijing, China) using Illumina HiSeqX Ten platforms. After quality control and trimming adaptor, reads were mapped onto human genome GRCh38. RNA-seq data have been deposited in the China National Center for Bioinformation (ID: PRJCA003751).

### Data Acquisition and Pre-Processing

Multiplatform genomics data was included in the study, including mRNA expression data, gene somatic mutation data, DNA copy data, and clinical information. For mRNA expression data, we collected the TCGA COAD AND READ datasets and three GEO datasets [GSE39582 (6), GSE17536 (15), and GSE14333 (16)] which meeting the following standard: samples were hybridized to the Affymetrix HGU133 Plus 2·0 (GPL570) platforms, each dataset contains more than 150 cases CRC patients, and information about the prognosis could be gathered. Besides, to analyze the efficiency of immunotherapy, we also included the “IMvigor” dataset using “IMvigor” package, which was generated from patients with metastatic urothelial cancer treated with anti-PD-L1 drugs (atezolizumab) (17). For TCGA mRNA datasets, the FPKM (fragments per kilobase of exon model per million reads mapped) normalized expression matrix was download form the Genomic Data Commons (GDC, https://portal.gdc.cancer.gov/). For microarray data, the raw “CEL” files were downloaded from GEO (http://www.ncbi.nlm.nih.gov/geo/) and subjected to a robust multiarray averaging method to perform background adjustment and quantile normalization using the “affy” packages (18). The corresponding clinical data was download at the same time. The gene somatic mutation data (MAF files) and DNA copy data (segment file) of TCGA COAD AND READ cohorts were download from GDC.

### Generation of EMT and DNA Repair Gene Panel and Unsupervised Clustering

EMT related genes were obtained from published studies and DNA repair related genes were obtained from Molecular Signatures Database (MSigDB) (4, 19, 20). Univariate cox regression was used to screening prognostic genes using GSE39582. Genes with a p-value less than 0.1 was selected for further analysis. Unsupervised clustering analysis was applied to identify characteristic expression patterns based on the expression of EMT and DNA repair gene panel, and patients were classified for further analysis. We use a consensus clustering algorithm to determine the number and stability of clusters (21). The “ConsensuClusterPlus” package was used to perform the above steps with 500 times repetitions to guarantee the stability of classification (22).

### Gene Set Variation Analysis (GSVA) and Functional Annotation

To investigate the biological pathways and processes enriched in different clusters, we applied GSVA which reckons the variation of pathway and bioprocess activity in the sample population by adopting unsupervised clustering method (23). The gene set files of “c2.cp.kegg.v6.2.symbols” and “c5.bp.v7.0.symbols” were downloaded from the MSigDB for running GSVA analysis using “GSVA” packages in R software. Adjusted P less than 0.05 was considered as statistically significance.

### Development and Validation of EMT and DNA Repair Risk Model

In order to reduce the dimension and pick the most meaningful prognostic indicators, we applied the least absolute shrinkage and selection operator (LASSO) Cox regression model to the EMT and DNA repair gene panel. LASSO is a penalized regression method that determines the regression coefficients by maximizing the log-likelihood function, while limiting the sum of the absolute values of the regression coefficients (24). The regression coefficients estimated by LASSO are sparse and many components are exactly zero. Thus, LASSO automatically deletes unnecessary covariates (25, 26). 10-fold cross validation was used to confirm the suitable tuning parameter (λ) for LASSO regression. The significant genes selected by LASSO were subsequently subjected to stepwise cox regression. The eventual regression model was selected based on the Akaike information criterion (AIC). GSE39582 cohort was served as the training set and the TCGA cohort was served as the validation set. A predicted value was calculated for every patient in the validation set on the basis of the risk model constructed in the training set. The ROC and AUC were used to assess the predictive discrimination ability of the risk model.

### Statistical Analysis

The statistically significant differences between non-normally distributed variables was analyzed by the Mann-Whitney U test, and normally distributed variables were reckoned adopting the unpaired Student’s t-test. In order to compare more than two groups, used Kruskal-Wallis as non-parametric methods, and adopted one-way ANOVA tests as parametric methods. Spearman and distance correlation analysis were used to calculate the correlation. The survival curves for the prognostic analysis were generated *via* the Kaplan-Meier method and log-rank tests were utilized to identify significance of differences. Use Cox proportional risk model and the “LR forward” stepwise approach to perform univariate and multivariate analyses. Evaluate the survival prediction of accuracy of the prognostic model *via* a time-related receiver operating characteristic curve (ROC) analysis. The R software (version 3.5.0) was used to conduct all statistical analyses, and all statistical P values were two-side, with p < 0.05 as statistically significance.

## Results

### Patient Characteristics and Prognostic Gene Identification

The patient characteristics contained in the datasets used in this study is summarized in **Table 1**. A total of 1,652 CRC patients from TCGA dataset and three GEO datasets (GSE39582, GSE17536, and GSE14333) were retrospectively analyzed in this study. Median age at diagnosis in different datasets ranged from 62 to 68 years. Male patients accounted for 54.48% (900/1652). EMT related genes were obtained from published studies (4, 20) and DNA repair related genes were obtained from MSigDB. We used GSE39582 as training set to identified prognostic gene. 98 genes (DNA repair: 41; EMT: 57) were eventually identified and defined as prognostic EMT and DNA repair genes for further study. Interestingly most of the EMT genes are epithelial markers, which were down-regulated in mesenchymal cells. Detailed information of the 98 genes was listed in **Supplemental Table 1**. The protein interaction network of the 98 genes were shown in **Supplemental Figure 1**.

**Table 1:** Baseline characteristics of patients in the discovery and validation cohorts.

Feature	GSE39582 cohort N=566 Number (%)	TCGA cohort N=619 Number (%)	GSE17536 cohort N=177 Number (%)	GSE14333 cohort N=290 Number (%)	All patients N=1652 Number (%)
Age					
Median (IQR)	66.91(17.00)	68.00(18.00)	66.00(18.00)	67.00(17.00)	–
Gender					
Male	310 (54.77)	330 (53.31)	96 (54.24))	164 (56.55)	900 (54.48)
Female	256 (45.23)	289 (46.69)	81 (45.76)	126 (43.45)	752 (45.52)
NA	0	0	0	0	0
T-stage					
Tis	3 (0.53)	1 (0.16)	–	–	4 (0.34)
T0	1 (0.18)	0 (0)	–	–	1 (0.08)
T1	11 (1.94)	20 (3.23)	–	–	31 (2.62)
T2	45 (7.95)	105 (16.96)	–	–	150 (12.66)
T3	367 (64.84)	422 (68.17)	–	–	789 (66.58)
T4	119 (21.02)	70 (11.31)	–	–	189 (15.95)
NA	20 (3.53)	1 (0.16)	–	–	21 (1.77)
N-stage					
N0	302 (53.36)	351 ((56.70)	–	–	653 (55.11)
N1	134 (23.67)	150 (24.23)	–	–	284 (23.97)
N2	98 (17.31)	115 (18.58)	–	–	213 (17.97)
N+	6 (1.06)	0 (0)	–	–	6 (0.51)
NA	26 (4.59)	3 (0.48)			29 (2.45)
M-stage					
M0	482 (85.16)	459 (74.15)	–	–	941 (79.41)
M1	61 (10.78)	87 (14.05)	–	–	148 (12.49)
NA	23 (4.06)	73 (11.79)	–	–	96 (8.10)
TNM				Dukes	–
0	4 (0.71)	0 (0)	0 (0)	A:44 (15.17)	–
I	33 (5.83)	105 (16.96)	24 (13.56)	B:94 (32.41)	–
II	264 (46.64)	227 (36.67)	57 (32.20)	C:91 (31.38)	–
III	205 (36.22)	179 (28.92)	57 (32.20)	D:61 (21.03)	–
IV	60 (10.60)	88 (14.22)	39 (22.03)		–
NA	0 (0)	20 (3.23)	0 (0)	0 (0)	–
MMR status					
dMMR	75 (13.25)	11 (1.78)	–	–	86 (7.26)
pMMR	444 (78.45)	105 (16.96)	–	–	549 (46.33)
NA	47 (8.30)	503 (81.26)	–	–	550 (46.41)

IQR, Interquartile range.

### Identification of Distinct Molecular Clusters Based on EMT and DNA Repair Genes

**Figure 1:**
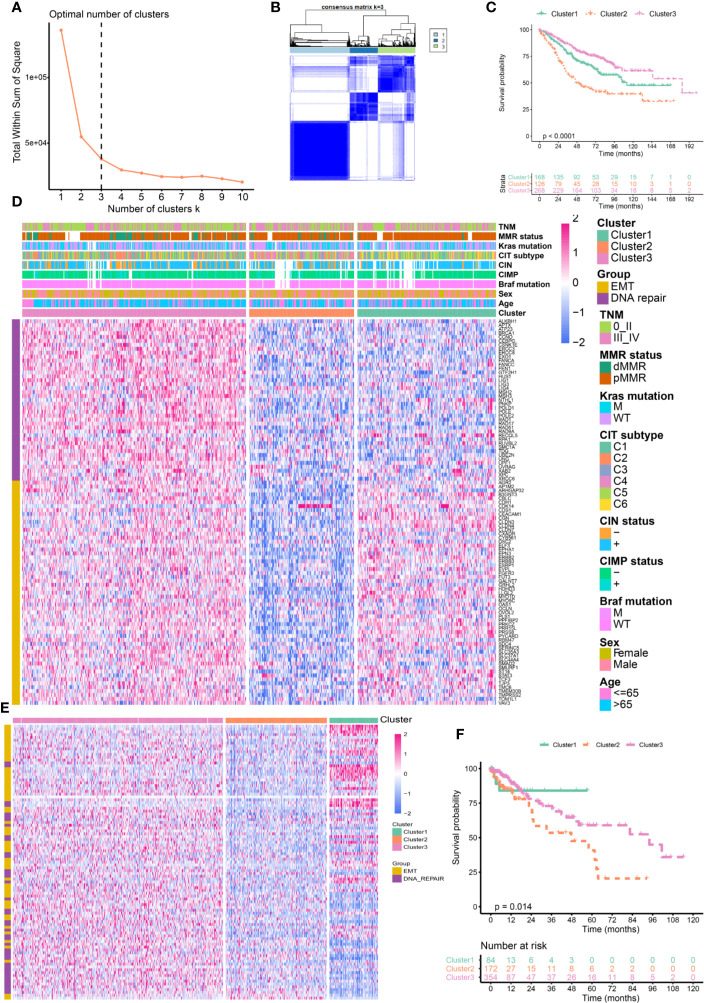
Identification of distinct molecular clusters based on epithelial mesenchymal transformation (EMT) and DNA repair genes. **(A)** The optimal number of clusters determined by Elbow method. **(B)** Consensus matrix for k = 3. **(C)** Overall survival of colorectal cancer (CRC) patients in the three clusters (GSE39582). **(D)** Heatmaps show the expression of 98 EMT and DNA repair genes (GSE39582). **(E)** Heatmaps show the expression of 98 EMT and DNA repair genes (TCGA). **(F)** Overall survival of CRC patients in the three clusters (TCGA).

We divided CRC samples in the GSE39582 into distinct molecular clusters according to 98 prognostic EMT and DNA repair genes. The optimal number of clusters was set at 3 (**Figure 1A**), as suggested by Elbow method. The consensus matrix heatmap revealed the identified three clusters (**Figure 1B**). It must be noted that the eventually incorporated EMT genes were principally epithelial cell markers whose expression levels negatively correlate with EMT. As shown in **Figure 1D**, CRC patients of different clusters possessed specific expression patterns of EMT and DNA repair genes. Cluster 1 (EPI^H^/DNA repair^L^) had increased expression of epithelial markers but down-regulated DNA repair genes. Cluster2 (EPI^L^/DNA repair^L^) was characterized by low expression of epithelial markers and DNA repair genes. Cluster3 (EPI^H^/DNA repair^H^) presented with apparent increased expression of epithelial markers and DNA repair genes. We selected recognized DNA repair genes (MLH1, MSH2, PMS1, and PMS2), which are key genes for determining MMR status and widely used in clinical practice (27), and epithelial genes (CDH1 and DSP) as well as mesenchymal genes (VIM, SNAI1, SNAI2, TWIST1, MMP2, and FN1) to analyze their expression among the three clusters (28). As shown in **Supplementary Figure 2**, the expression of DNA repair genes (MLH1, MSH2, PMS1, and PMS2) and epithelial genes (CDH1 and DSP) were significantly increased in the Cluster 3(EPI^H^/DNA repair^H^) while significantly decreased in the Cluster 2(EPI^L^/DNA repair^L^). The expression of mesenchymal genes (VIM, SNAI1, SNAI2, TWIST1, MMP2, and FN1) were significantly decreased in the Cluster 3(EPI^H^/DNA repair^H^) but increased in the Cluster 2(EPI^L^/DNA repair^L^). These results indicated that DNA repair was active but the EMT was suppressive in Cluster 3, which contrasts with gene expression pattern in Cluster 2. The three Cluster had different survival profiles, with the Cluster 3 having the best prognosis but Cluster 2 having the worst prognosis (**Figure 1C**).

We further validated the 98 genes panel in independent cohort. The first cohort was from TCGA comprised 619 cases of CRC. Three distinct molecular clusters were identified as described above (Cluster 1 (EPI^H^/DNA repair^L^), Cluster 2(EPI^L^/DNA repair^L^), and Cluster 3(EPI^H^/DNA repair^H^), **Figure 1E**). Survival analysis confirmed that cluster have distinct outcomes. Here again, cluster 2 having the worst prognosis (**Figure 1F**). The second cohort was from GSE14333 receive adjuvant chemotherapy. As shown in **Supplementary Figure 3A**, three distinct molecular clusters were identified and Cluster 2 having the worst prognosis (**Supplementary Figure 3B**). The third validation cohort was from GSE17536 comprised 177 cases of CRC. We also identified three distinct molecular clusters as described above (**Supplementary Figure 3C**). Kaplan–Meier analysis revealed that the three subgroups have distinct outcome, that the Cluster 2 had the worst prognosis while Cluster 1 and Cluster 3 had similar outcome (**Supplementary Figure 3D**).

### Correlation of the Clusters With Clinical Characteristics and Classical Classification

**Figure 2:**
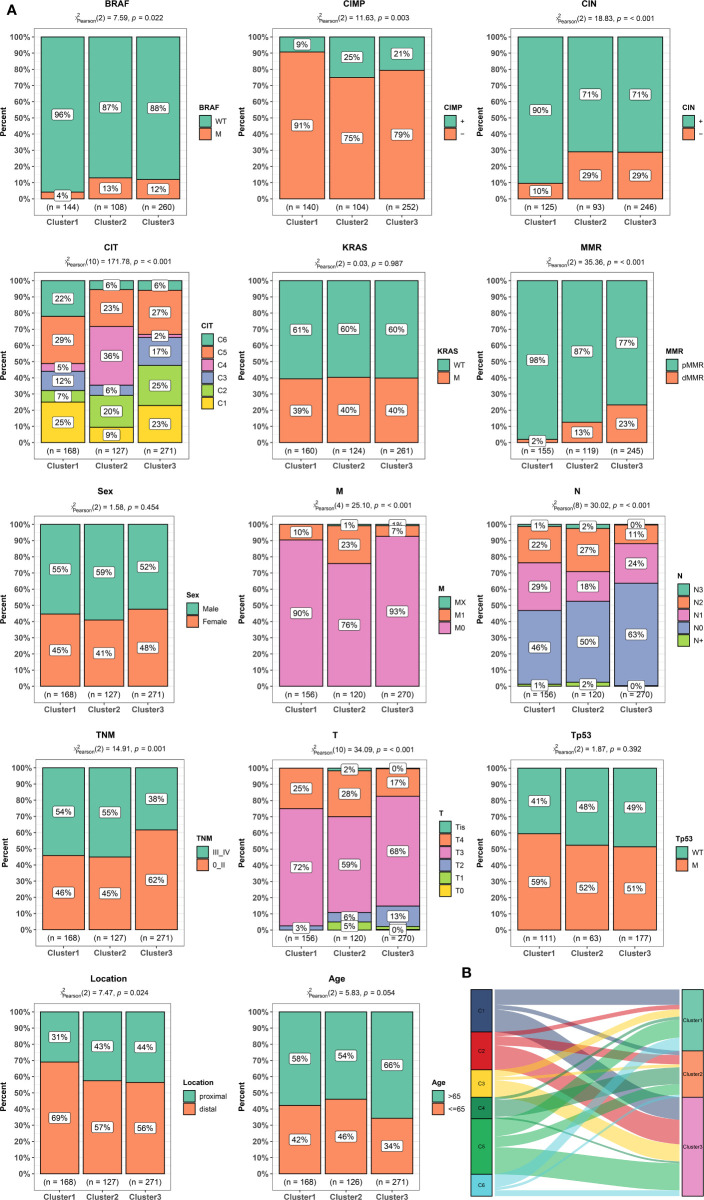
Clinical and molecular characteristics of colorectal cancer (CRC) patients according to the cluster. **(A)** Bar plots showing the proportion of gender, age, stage, tumor localization, KRAS, BRAF, and TP53 mutations, hypermutated phenotype, mismatch repair status (MMR), CpG island methylator phenotype (CIMP), chromosomal instability (CIN), and Cartes d’Identite´ des Tumeurs (CIT) subtypes in different clusters. **(B)** Sankey chart displaying the distribution of C1–C6 CIT subtypes in different clusters.

The relationships between CRC classifications and clinical characteristics were then investigated by using the GSE39582 (**Figure 2A** and **Supplementary Table 2**). Cluster 1 was associated with lower proportion of BRAF mutation, CpG island methylator phenotype (CIMP) and dMMR. But Cluster 1 has a higher proportion of patients with distal CRC, lymphatic metastasis as well as CIN and mainly enriched in C1, C5, and C6 of CIT subtype. Cluster 2 was associated with high percentage of BRAF mutation, CIMP, T4 stage, distant metastasis, and young patients. Cluster 2 was mainly enriched in C4 of CIT subtype. Cluster 3 had a high percentage of dMMR, node-negative, no distant metastasis and elderly patient. Cluster 3 was mainly concentrated in the C2, C3, and C5 of CIT subtype. **Figure 2B** summarized the relationship between CLT subtype and different clusters. There was no significant difference in the distribution of KRAS mutation, Tp53 mutation and gender among different clusters. We further validated the association by using TCGA dataset. As shown in **Supplementary Figure 4**, We again found that Cluster 2 was associated with a higher proportion of T4 and stage III–IV. But, node-negative CRC and patients without lymphatic invasion (LV) and vessel invasion (VL) have higher percentage in Cluster 3.

### Characteristics of Tumor Genome Variation in Different Clusters

**Figure 3:**
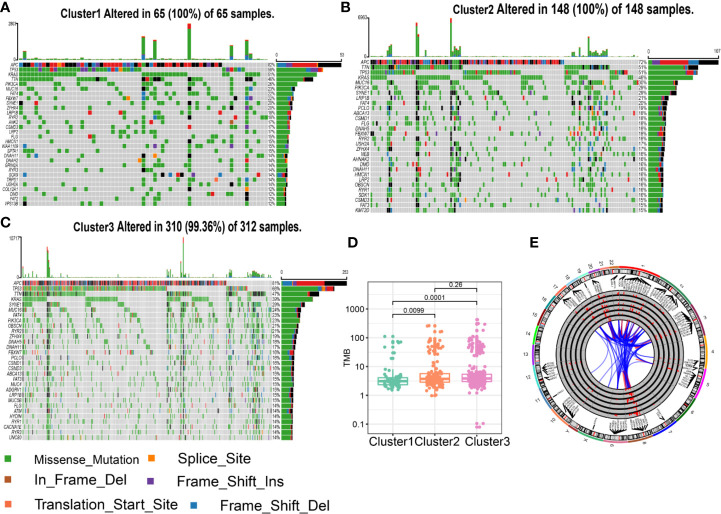
Characteristics of tumor genome variation in different clusters. **(A)** Genes with top 30 mutation frequency in Cluster1. **(B)** Genes with top 30 mutation frequency in Cluster2. **(C)** Genes with top 30 mutation frequency in Cluster3. **(D)** Tumor mutation load (TMB) level in different clusters. **(E)** Circular visualization of the copy number variant (CNV) alteration in each cluster. The outermost circle shows the location of the CNV gene. The histogram shows the frequency of CNV in in different clusters. From outside to inside: genes significant deletion in Cluster1, significant amplification in Cluster1, significant deletion in Cluster2, significant amplification in Cluster2, significant deletion in Cluster3, significant amplification in Cluster3. The link lines dedicated gene interactions.

TCGA has completed a comprehensive molecular characterization of CRC, thus we analyzed the distribution differences of somatic single nucleotide variants (SNVs) among different clusters based on TCGA dataset. As shown in **Figures 3A–C**, the top three genes with the highest frequency of mutations in cluster1 were APC (82%), TP53 (58%), and KRAS (51%), and those in Cluster2 are APC (72%), TTN (51%), and TP53 (51%), and those in Cluster3 are APC (81%), TP53 (66%), and TTN (47%). There was no significant difference in the frequency of somatic mutations in the three clusters. Tumor mutation burden (TMB) is a measurement of somatic mutation carried by cancer cells and high TMB status presented a durable clinical response to anti-PD-1/PD-L1 immunotherapy in CRC (29). We compared the TMB among different clusters. as shown in **Figure 3D**, the Cluster2 and 3 had the highest TMB while the Cluster1 had the lowest TMB. These results indicated that Cluster2 and 3 might benefit from immunotherapy. Copy number variants (CNVs) are a key component of genetic variation and have a greater impact in the genome than SNVs. We investigated alteration frequency of CNVs among different clusters. A total of 352 genes with significant differences in amplification frequency or deletion frequency among the three clusters were identified. The genes location, amplification frequency and deletion frequency in each cluster was summarized in **Figure 3E**. **Supplementary Figure 5** presented representative genes with significant differences in amplification frequency or deletion frequency among the three clusters. We performed gene enrichment analysis to explore biological processes and pathways involved in aberrant amplification or deletion of genes (**Supplementary Figure 6**). Genes significantly amplified in the Cluster3 were enriched in Defense response to bacterium and Focal adhesion, which indicated that Cluster3 might associate with immune and metastasis. Pathways enrichment analysis suggested that significantly amplified genes in Cluster2 were enriched in Cell cycle and Cell adhesion molecules, indicated that Cluster2 might associate with cell proliferation and metastasis.

### Clusters Predicts Therapeutic Benefit of Chemotherapy and Immunotherapy

**Figure 4:**
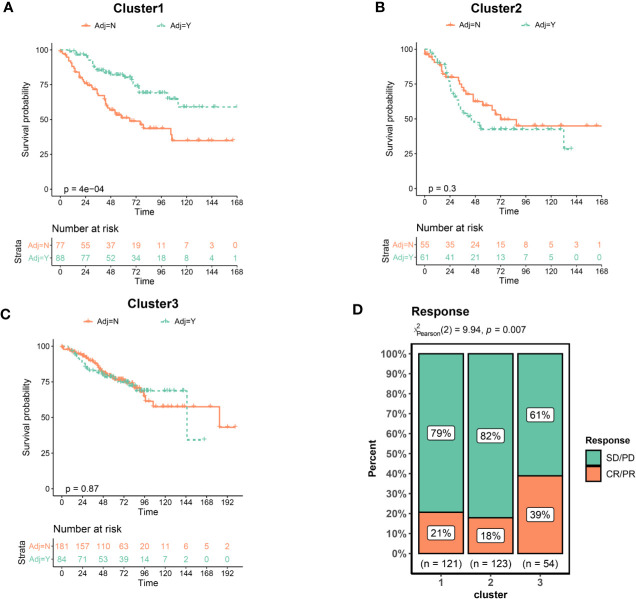
Clusters predicts therapeutic benefit of chemotherapy and immunotherapy. **(A)** Kaplan–Meier curves of overall survival for patients in Cluster1 stratified by receipt of adjuvant chemotherapy. **(B)** Cluster2. **(C)** Cluster3. **(D)** Response rate of patients to immunotherapy. CR, complete response; PR, partial response; SD, stable disease; PD, progressive disease.

Adjuvant chemotherapy (ADJC) is the primary treatment strategy for patients with non-metastatic CRC cancer (30). Given that the GSE39582 dataset provided information on chemotherapy in patients, we utilized this dataset to analyze the relationship between EMT and DNA repair gene clusters and ADJC benefit. We used OS to assess treatment outcome. Interestingly, only patients in the Cluster 1 had improved OS after receiving ADJC (**Figure 4A**). No significant difference in OS of patients in Cluster 2 and 3 regardless of whether they received ADJC (**Figures 4B, C**). These results indicated that patients in the Cluster 1 might benefit from chemotherapy.

Immunotherapy has recently emerged as an effective new therapy for CRC. However, immunotherapy is currently indicated only for CRC patients with dMMR, which only account for about 5%–15%. It is crucial to identify CRC patients benefit from immunotherapy. We collected an immunotherapy data set (Imvigor210) to explore whether the clusters could predict the immune treatment benefit. As shown in **Figure 4D**, the proportion of patients achieved a complete response (CR) or partial response (PR) was significantly increased in the Cluster3. These results indicated that patients in the Cluster 3 benefited from immunotherapy at a higher rate.

### Biological Pathways and Processes Enriched in Different Clusters

**Figure 5:**
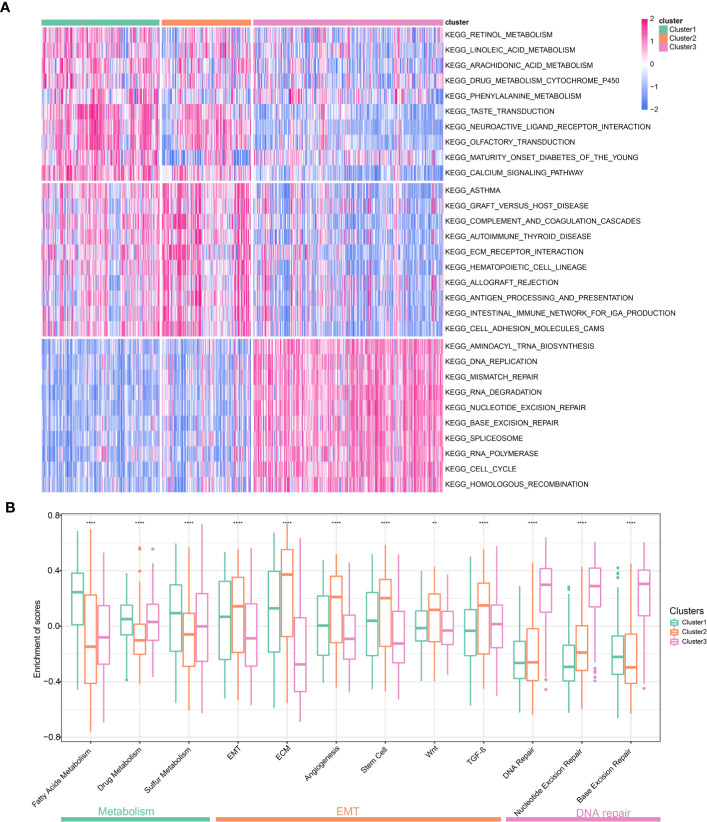
Biological pathways and processes enriched in different Clusters. **(A)** Heatmap of different pathways among the three clusters. Each cluster exhibit 10 of the most distinctive KEGG pathways. **(B)** Comparison representative pathways and its enrichment scores in different clusters.

To explore the biological characteristics among these distinct clusters, we performed GSVA enrichment analysis. It should be noted that this was a pathway-level comparison for exploring the biological significance behind the different clusters. It was not a re-phenotyping using a new set of genes. The enrichment analysis results of KEGG pathway showed that Cluster1 was markedly enriched in metabolic pathways such as Retinol Metabolism, Linoleic acid Metabolism, and Arachidonic acid Metabolism (**Figure 5A**). Cluster2 presented enrichment pathways associated with EMT including ECM receptor interactions and Cell adhesion molecules (CAMs). While Cluster3 was prominently related to DNA repair pathways such as DNA Replication, Mismatch Repair and Base excision Repair. **Figure 5B** presented representative pathways and its enrichment scores in different clusters. Again, metabolic pathways had the highest enrichment scores in the Cluster1 and EMT related pathways including extracellular matrix (ECM), Wnt pathways, and TGF-β pathways had the highest enrichment scores in the Cluster2. DNA repair pathways had the highest enrichment scores in the Cluster3. The enrichment scores for the above pathways were significantly different (all P <0.05, **Figure 5B**). We further explored biological processes enriched in distinct clusters. Different clusters had characteristic biological processes (**Supplementary Figure 7**). Biological processes associated with Amino acid transport, Ion transport and Transmission of neural signal were significantly enriched in Cluster1 (**Supplementary Figure 8A**). Cluster2 were enriched in Mesenchymal formation, Immune response and Amino acid transport (**Supplementary Figure 8B**). Besides, biological processes significantly enriched in Cluster3 including RNA processing and DNA repair (**Supplementary Figure 8C**). Based on the above analyses, we were surprised to learn that three clusters had significantly distinct biological characteristics. Cluster1 was characterized by activation of metabolic pathways and Cluster2 was characterized by EMT activation. Cluster3 was characterized by activation of DNA repair.

### Construction of EMT and DNA Repair Risk Scores Related to Prognosis and Treatment Response

**Figure 6:**
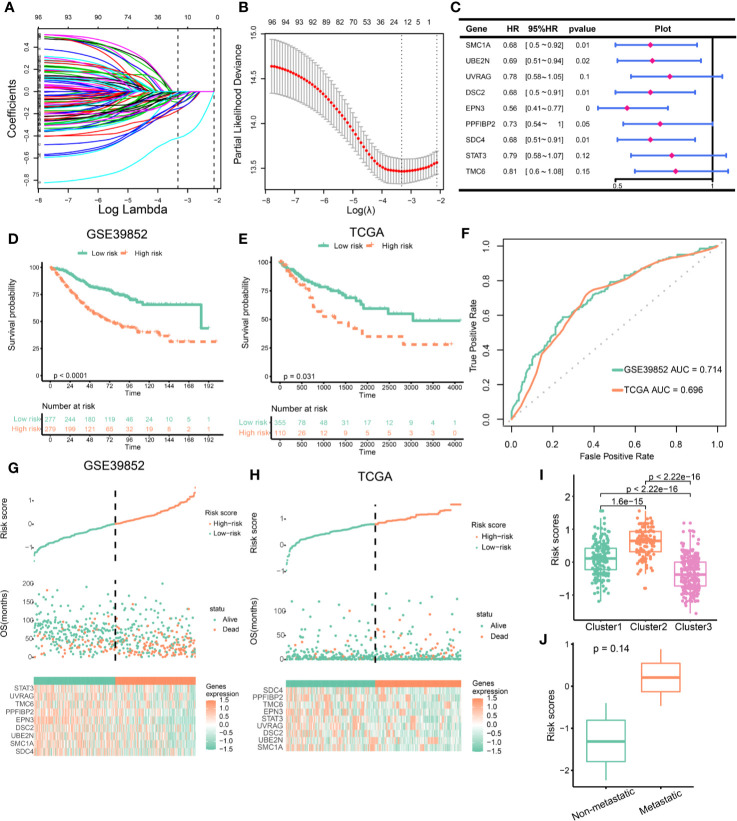
Construction of epithelial mesenchymal transformation (EMT) and DNA repair risk scores. **(A)** LASSO regression coefficient profiles of 98 EMT and DNA repair genes. **(B)** Tuning parameter (λ) selection in the LASSO regression used 10-fold-cross-validation *via* minimum criteria. The black vertical lines are plotted at the optimal λ based on the minimum criteria and 1 standard error for the minimum criteria. **(C)** The hazard ratios and p-values of the 9 genes in the risk model. **(D)** Kaplan–Meier curves of overall survival for patients in GSE39582 stratified by risk scores. **(E)** Kaplan–Meier curves of overall survival for patients in TCGA stratified by risk scores. **(F)** The ROC curves for the risk model in GSE39582 and TCGA. **(G, H)** Construction and analysis of risk scores. The top panels indicate the risk scores of the patients. The middle panels depict the survival statuses and survival times of the patients distributed by risk score. The bottom panels display the heatmap of the expression for the nine genes distributed by risk score. **(G)** GSE39582 cohort; **(H)** TCGA cohort. **(I)** Comparison of risk scores across clusters. **(J)** Risk scores in metastatic VS. non-metastatic colorectal cancer (CRC) patients.

To develop clinically useful prognostic and efficacy assessment models for individual, we applied the LASSO Cox regression model to the 98 EMT and DNA repair genes for dimension reduction. GSE39582 cohort was served as training set and TCGA cohort were served as validation cohort. As shown in **Figures 6A, B**, the most appropriate tuning parameter λ for LASSO Cox regression analysis was determined to be 0.036 when the partial likelihood deviance was the smallest. The 16 genes with non-zero coefficients in the tuning parameter were selected and subject to stepwise cox regression. Ultimately, nine genes were used to constructed the scoring system. The hazard ratios and P-values of the nine genes in the scoring model were summarized in **Figure 6C**. We compared the expression of nine genes in different clusters, and interestingly, these nine genes were significantly differentially expressed in different clusters (**Supplementary Figure 9**), suggesting that these genes represent characteristics of different clusters. Patients were divided into high-risk and low-risk groups according to the risk score predicted. And survival analysis demonstrated that the EMT and DNA repair risk scores had significant power to distinguish good from poor outcomes in CRC patients (P<0.001) (**Figure 6D**). We further validated the scoring model in TCGA cohort. Patients with high-risk had worse outcomes compared with low-risk (**Figure 6E**). ROC curve analysis revealed that the EMT and DNA repair risk scores had similar degree of discrimination in GSE39582 cohort and TCGA cohort (GSE39582: AUC= 0.714; TCGA: AUC=0.696, **Figure 6F**). The correlation between risk scores, gene expression and survival state were present in the **Figures 6G, H**. Next, we analyzed the association between risk scores and cluster. The Cluster 3, with a better prognosis, had the lowest risk score, while Cluster2, with the worst prognosis, had the highest risk score. And Cluster1, with intermediate prognosis, had medium risk score (**Figure 6I**). We further validate the risk scores using in-house data. We found that patients with metastatic CRC had higher risk scores than patients with non-metastatic CRC, but the difference was not statistically significant, possibly because of the small sample size (**Figure 6J**). These results indicated that the risk scores were closely related to prognosis and different clusters had distinct risk scores.

Since the EMT and DNA repair genes clusters were associated with immunotherapeutic response, we investigated whether the risk scores can predict immunotherapeutic benefit. Cluster 3 benefited from immunotherapy at a higher rate. We first compared the levels of risk scores in different clusters based on Imvigor210 cohort. Cluster 3 had lowest risk scores, which indicated that low risk scores predicated immunotherapeutic benefit (**Supplementary Figure 10A**). Besides, the proportion of CR or PR was significantly increased in patients with low risk (**Supplementary Figure 10B**). In patients receiving immunotherapy, patients with low risk had better prognosis than those with high risk (**Supplementary Figure 10C**). These findings suggested that low risk scores predicated immunotherapeutic benefit.

## Discussion

With the development of research, we gain a deeper understanding of the biological and molecular characteristics of CRC (31). CRC classification based on characteristic pathways may be a promising way to simplify the classification process and improve clinical utility. Activation of EMT pathways is associated with malignant behavior and drug resistance (32). While activation of DNA repair pathways is a key feature of “hot tumor” and a predictor of immunotherapy (33). In the present study, we identified three distinct CRC clusters based on a combined EMT and DNA repair gene panel.

**Figure 7:**
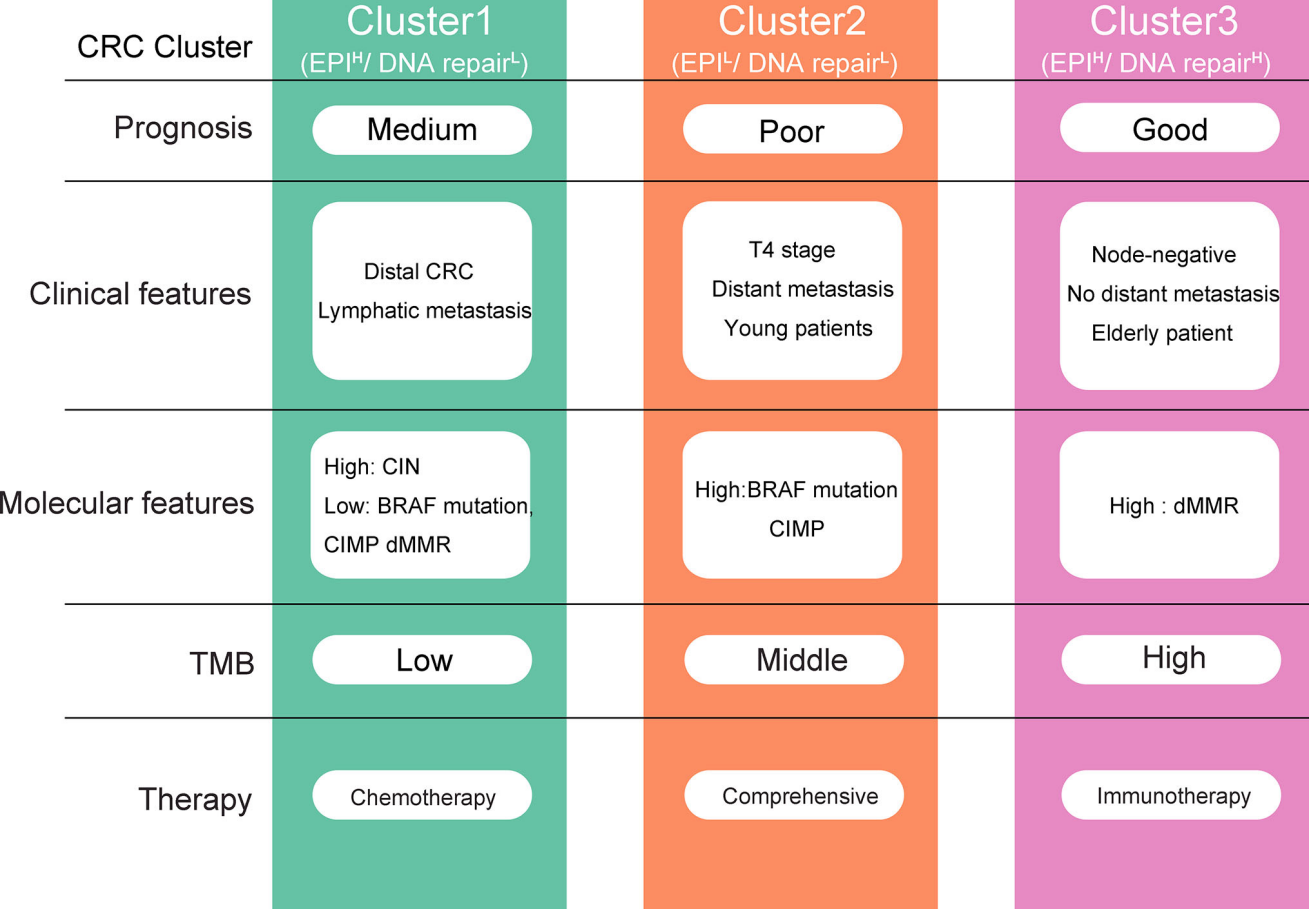
Overview of the characteristics of three colorectal cancer (CRC) clusters. EPI, Epithelial.

The three CRC clusters differ significantly in clinical characteristics, prognosis, genomic variation, active pathways, and response to chemotherapy and immunotherapy (**Figure 7**). Clustet1 (EPI^H^/DNA repair^L^) was characterized by down-regulated DNA repair pathways but active epithelial markers and metabolism pathway. Clustet1 has intermediate prognosis and lower proportion of BRAF mutation, CpG island methylator phenotype (CIMP) and dMMR. But Cluster1 has a higher proportion of patients with distal CRC as well as lymphatic metastasis. TMB scores was the lowest in the Cluster1. Patients in the Cluster1 might benefit from chemotherapy but not immunotherapy. Besides, Cluster1 was associated with a moderate EMT and DNA repair risk scores. The Cluster2 (EPI^L^/DNA repair^L^) was characterized by down-regulated DNA repair and epithelial markers. Clustet2 was associated with the worst prognosis. Cluster 2 has a high percentage of BRAF mutation, CIMP, T4 stage, distant metastasis, and young patients. Clustet2 presented with high TMB and genes significantly amplified in Cluster2 were enriched in Cell cycle and Cell adhesion molecules. Patients in the Cluster2 might not benefit from chemotherapy and immunotherapy. The EMT and DNA repair risk scores was the highest in the Cluster2. The Cluster3 (EPI^H^/DNA repair^H^) presented with activation of DNA repair pathway and epithelial markers. Patients in Cluster3 had the best prognosis. Cluster 3 had a high percentage of dMMR, node-negative, no distant metastasis, or LV or VL and elderly patient. Clustet3 presented with high TMB and genes significantly amplified in Cluster3 were enriched in Defense response to bacterium and Focal adhesion. Cluster 3 benefited from immunotherapy at a higher rate. The EMT and DNA repair risk scores was the lowest in the Cluster3. Therefore, the identification of three different clusters is of great significance for the accurate treatment of CRC.

Chemotherapy is one of the main treatment strategies for CRC, which is critical for creating surgical opportunities and preventing tumor recurrence (34). Detecting patients who may benefit from chemotherapy is an important step in precision treatment. Activation of EMT is a recognized factor in the induction of chemotherapy resistance (35). 5-fluorouracil (5-Fu) based chemotherapy is commonly used in convention chemotherapy of CRC (36). The 5-Fu resistance is partially induced by EMT *via* the Akt gene or mediated by Twist, miR-200c, miR-141 (26, 34). Besides, down-regulation of EMT-related miR-200c and miR-141 could induced resistance to oxaliplatin, which is one of the most common drugs in CRC chemotherapy (37). Moreover, EMT is strongly associated with tumor proliferation, infiltration, metastasis, tumor budding (10). Given that Cluster2 presents with activation of EMT, we have reasons to infer that Cluster2 has a poor prognosis and does not benefit from chemotherapy.

Metabolic reprogramming is a hallmark of malignancy (38). To support the rapid proliferation, progression, and metastasis, cancer cells rewire metabolic pathways *via* increased generation of adenosine triphosphate (ATP), macromolecule synthesis, and antioxidant regeneration (39). Abnormal metabolic pathways provide new targets for the treatment of cancer and sensitize cancer chemotherapy (40). For example, increased expression of MUC1 enhanced glycolysis, nonoxidative PPP, and pyrimidine biosynthesis (41). Inhibition of MUC1 sensitizes cancer cell lines to 5-FU (24, 42). Combination of antimetabolic therapy and chemotherapy may yield better response rates (43). Based on our analysis, Cluster1 present with increased metabolism pathways, we speculated that Cluster1 patients may benefit from anti-metabolic therapy and chemotherapy.

Currently, benefits of immunotherapy have received immense research interest because of the impressive long-lasting response seen in several solid tumors (33, 44). In CRC, immune response and survival benefit were limited to mismatch-repair-deficient and microsatellite instability-high (dMMR–MSI-H) CRC patients, who account for only a small percentage of CRC patients (around 8%–15%) (3, 45). Thus, the selection criteria for candidates who are likely to benefit from such regimens requires further investigation. In the present study, we found that patients in the Cluster3 had the highest response rate to immunotherapy (around 40%). Besides, Cluster3 was present with high proportion of dMMR and TMB, which were recognized immunotherapeutic response prediction marker. We infer that patients in Cluster3 may benefit from immunotherapy. In addition, an interesting phenomenon we found was that although Cluster3 had a higher proportion of dMMR, the expression of key MMR genes was elevated. The MMR gene expression products are called MMR proteins, and they exist as heterodimeric complexes for mismatch base identification and subsequent repair (45). Most mutations in the MMR gene interfere with dimerization, leading to proteolytic degradation of the heterodimer, resulting in the loss of obligatory and secondary proteins (27). This assumption may explain why mRNA is elevated but protein expression is down-regulated. Further research is needed to confirm this assumption.

In recent years, the availability of clinical-grade, rapid, and inexpensive benchtop next-generation sequencers, as well as prepackaged analytical software and reagents, has driven the rapid growth and popularity of gene panel assays in clinical laboratories (46). The gene panel amplifies only specific genes and therefore has the advantage of lower cost and faster speed (47). The limitations of gene panel assay are the high investment in equipment and the cost of sequencing reagents, making it impractical in the case of too small a total specimen volume. In addition, despite the wide application of the technology in recent years, there is still a shortage of experienced professionals. This lack of expertise results in variable quality of analysis and interpretation of the complex data. It is also unclear how to validate, control and charge for these tests, limiting their deployment in hospital laboratories (48).

This study has some limitations. First, the patient population is heterogeneous due to the retrospective nature of this study. Second, the robustness of the predictive value of the gene panel needs further validation in large prospective clinical trials. Third, experimental studies are needed to further elucidate the biological significances of the gene panel. Fourth, although our proposed EMT and DNA repair gene panel has potential clinical applications, such as the development of molecular typing kits for colorectal cancer, many issues remain unresolved, such as further identification of target genes, design of probes and determination of expression thresholds.

## Conclusion

In conclusion, the present study developed and validated a combined EMT and DNA repair gene panel for CRC classification. Three CRC clusters with distinct characteristics were identified. This gene panel may have clinical application for prognosis estimation and guiding chemotherapy as well as checkpoint inhibitors.

## Data Availability Statement

The original contributions presented in the study are included in the article/**Supplementary Material**. Further inquiries can be directed to the corresponding authors.

## Ethics Statement

The studies involving human participants were reviewed and approved by The Ethics and Human Subject Committee of Guangxi Medical University Cancer Hospital. The patients/participants provided their written informed consent to participate in this study.

## Author Contributions

Conceived and designed the experiments: XH, JL, HL, XM, YZ, and WT. Performed the data collection: XH, JL, HL, LZ, YD, and YM. Analyzed the data: XH, JL, HL, XM, YZ, LZ, YD, and YM. Contributed reagents/materials/analysis tools: HL, XM, YZ, and WT. Contributed to the writing of the manuscript: XH, JL, HL, XM, YZ, WT, LZ, YD, and YM. All authors contributed to the article and approved the submitted version.

## Funding

This study was funded by the 2019 Guangxi University High-level Innovation Team and the Project of Outstanding Scholars Program, and Guangxi Science and Technology Project (2019AC03004); Guangxi Clinical Research Center for Colorectal Cancer (Guike: AD19245197).

## Conflict of Interest

The authors declare that the research was conducted in the absence of any commercial or financial relationships that could be construed as a potential conflict of interest.

## Supplementary Material

Supplementary Figure 1 Protein-protein interaction (PPI) network of the 98 EMT and DNA repair genes. Genes belonging to EMT are represented in red and Genes belonging to DNA repair are represented in blue. The size of a gene is positively correlated with the number of genes it links.

Supplementary Figure 2 Expression of representative EMT and DNA repair genes in the three CRC clusters. DNA repair genes were marked red and epithelial genes were green. Mesenchymal genes were orange.

Supplementary Figure 3 Identification of distinct molecular clusters based on EMT and DNA repair genes using GSE14333 and GSE17536. **(A)** Heatmaps show the expression of 98 EMT and DNA repair genes (GSE14333). **(B)** Survival analysis of CRC patients in the three clusters (GSE14333). **(C)** Heatmaps show the expression of 98 EMT and DNA repair genes (GSE17536). D: Survival analysis of CRC patients in the three clusters (GSE17536).

Supplementary Figure 4 Clinical characteristics of colorectal cancer (CRC) patients according to the cluster (TCGA data). Bar plots showing the proportion of tumor stage, tumor localization, lymphatic invasion (LV) and vessel invasion (VL) in different clusters.

Supplementary Figure 5 Representative genes with significant differences in amplification or deletion frequency among the three clusters.

Supplementary Figure 6 Enrichment analysis of aberrant amplification or deletion of genes. Showing the top five terms with a P value less than 0.05. Left panel: GO biological process; Right panel: KEGG pathways.

Supplementary Figure 7 Heatmap of different biological processes among the three clusters. Each cluster exhibit 30 of the most distinctive GO biological processes.

Supplementary Figure 8 Tree diagram of distinctive GO biological processes in each cluster. Clustering of GO terms according to the common genes contained in the different terms. The closer the two terms are, the more genes they share. **(A)** Cluster1; **(B)** Cluster2; **(C)** Cluster3.

Supplementary Figure 9 The expression of 9 prognostic genes in different clusters.

Supplementary Figure 10 Correlation between risk score and immunotherapy response. **(A)** Comparison of risk scores across clusters based on “IMvigor” dataset. **(B)** Response rate of patients to immunotherapy. Patients were stratified according the risk scores. CR, complete response; PR, partial response; SD, stable disease; PD, progressive disease. **(C)** Kaplan–Meier curves for patients receiving immunotherapy.

## References

[1] Siegel RL Miller KD Jemal A Cancer statistics, 2020. CA Cancer J Clin (2020) 70:7–30. 10.3322/caac.21590 31912902

[2] Edge SB Compton CC The American Joint Committee on Cancer: the 7th Edition of the AJCC Cancer Staging Manual and the Future of TNM. Ann Surg Oncol (2010) 17:1471–4. 10.1245/s10434-010-0985-4 20180029

[3] Huang X Liu J Wu G Chen S Pc FJ Xie W Development and Validation of a Nomogram for Preoperative Prediction of Perineural Invasion in Colorectal Cancer. Med Sci Monit (2019) 25:1709–17. 10.12659/MSM.914900 PMC6415589 30837449

[4] Piskol R Huw L Sergin I Kljin C Modrusan Z Kim D A Clinically Applicable Gene-Expression Classifier Reveals Intrinsic and Extrinsic Contributions to Consensus Molecular Subtypes in Primary and Metastatic Colon Cancer. Clin Cancer Res (2019) 25:4431–42. 10.1158/1078-0432.CCR-18-3032 31004000

[5] Roelands J Kuppen PJK Vermeulen L Maccalli C Decock J Wang E Immunogenomic Classification of Colorectal Cancer and Therapeutic Implications. Int J Mol Sci (2017) 18:2229. 10.3390/ijms18102229 PMC5666908 29064420

[6] Marisa L de Reynies A Duval A Selves J Gaub MP Vescovo L Gene expression classification of colon cancer into molecular subtypes: characterization, validation, and prognostic value. PloS Med (2013) 10(5):e1001453. 10.1371/journal.pmed.1001453 23700391 PMC3660251

[7] Sorlie T Tibshirani R Parker J Hastie T Marron JS Nobel A Repeated observation of breast tumor subtypes in independent gene expression data sets. Proc Natl Acad Sci USA (2003) 100(14):8418–23. 10.1073/pnas.0932692100 PMC166244 12829800

[8] Christiansen JJ Rajasekaran AK Reassessing epithelial to mesenchymal transition as a prerequisite for carcinoma invasion and metastasis. Cancer Res (2006) 66:8319–26. 10.1158/0008-5472.CAN-06-0410 16951136

[9] Lu W Kang Y Epithelial-Mesenchymal Plasticity in Cancer Progression and Metastasis. Dev Cell (2019) 49:361–74. 10.1016/j.devcel.2019.04.010 PMC6506183 31063755

[10] Cao H Xu E Liu H Wan L Lai M Epithelial-mesenchymal transition in colorectal cancer metastasis: A system review. Pathol Res Pract (2015) 211(8):557–69. 10.1016/j.prp.2015.05.010 26092594

[11] Feng YL Chen DQ Vaziri ND Guo Y Zhao YY Small molecule inhibitors of epithelial-mesenchymal transition for the treatment of cancer and fibrosis. Med Res Rev (2020) 40(1):54–78. 10.1002/med.21596 31131921

[12] Mota MBS Carvalho MA Monteiro ANA Mesquita RD DNA damage response and repair in perspective: Aedes aegypti, Drosophila melanogaster and Homo sapiens. Parasit Vectors (2019) 12(1):533. 10.1186/s13071-019-3792-1 31711518 PMC6849265

[13] Chae YK Anker JF Oh MS Bais P Namburi S Agte S Mutations in DNA repair genes are associated with increased neoantigen burden and a distinct immunophenotype in lung squamous cell carcinoma. Sci Rep (2019) 9(1):3235. 10.1038/s41598-019-39594-4 30824826 PMC6397194

[14] Le DT Uram JN Wang H Bartlett BR Kemberling H Eyring AD PD-1 Blockade in Tumors with Mismatch-Repair Deficiency. N Engl J Med (2015) 372(26):2509–20. 10.1056/NEJMoa1500596 PMC4481136 26028255

[15] Williams CS Bernard JK Demory Beckler M Almohazey D Washington MK Smith JJ ERBB4 is over-expressed in human colon cancer and enhances cellular transformation. Carcinogenesis (2015) 36(7):710–8. 10.1093/carcin/bgv049 PMC4572918 25916654

[16] Jorissen RN Gibbs P Christie M Prakash S Lipton L Desai J Metastasis-Associated Gene Expression Changes Predict Poor Outcomes in Patients with Dukes Stage B and C Colorectal Cancer. Clin Cancer Res (2009) 15(24):7642–51. 10.1158/1078-0432.CCR-09-1431 PMC2920750 19996206

[17] Mariathasan S Turley SJ Nickles D Castiglioni A Yuen K Wang Y TGFbeta attenuates tumour response to PD-L1 blockade by contributing to exclusion of T cells. Nature (2018) 554(7693):544–8. 10.1038/nature25501 PMC6028240 29443960

[18] Gautier L Cope L Bolstad BM Irizarry RA affy–analysis of Affymetrix GeneChip data at the probe level. Bioinformatics (2004) 20(3):307–15. 10.1093/bioinformatics/btg405 14960456

[19] Subramanian A Tamayo P Mootha V Mukherjee S Ebert B Gillette M Gene set enrichment analysis: a knowledge-based approach for interpreting genome-wide expression profiles. Proc Natl Acad Sci USA (2005) 102(43):15545–50. 10.1073/pnas.0506580102 PMC1239896 16199517

[20] Kardos J Chai S Mose LE Selitsky SR Krishnan B Saito R Claudin-low bladder tumors are immune infiltrated and actively immune suppressed. JCI Insight (2016) 1(3):e85902. 10.1172/jci.insight.85902 27699256 PMC5033914

[21] Nidheesh N Abdul Nazeer KA Ameer PM An enhanced deterministic K-Means clustering algorithm for cancer subtype prediction from gene expression data. Comput Biol Med (2017) 91:213–21. 10.1016/j.compbiomed.2017.10.014 29100115

[22] Wilkerson MD Hayes DN ConsensusClusterPlus: a class discovery tool with confidence assessments and item tracking. Bioinformatics (2010) 26:1572–3. 10.1093/bioinformatics/btq170 PMC2881355 20427518

[23] Hanzelmann S Castelo R Guinney J GSVA: gene set variation analysis for microarray and RNA-seq data. BMC Bioinf (2013) 14:7. 10.1186/1471-2105-14-7 PMC3618321 23323831

[24] Huang X Liu J Mo X Liu H Wei C Huang L Systematic profiling of alternative splicing events and splicing factors in left- and right-sided colon cancer. Aging (Albany NY) (2019) 11(19):8270. 10.18632/aging.102319 31586988 PMC6814588

[25] Goeman JJ L1 penalized estimation in the Cox proportional hazards model. Biom J (2010) 52:70–84. 10.1002/bimj.200900028 19937997

[26] Liu J Huang X Yang W Li C Li Z Zhang C Nomogram for predicting overall survival in stage II-III colorectal cancer. Cancer Med (2020) 9(7):2363–71. 10.1002/cam4.2896 PMC7131840 32027098

[27] Luchini C Bibeau F Ligtenberg MJL Singh N Nottegar A Bosse T ESMO recommendations on microsatellite instability testing for immunotherapy in cancer, and its relationship with PD-1/PD-L1 expression and tumour mutational burden: a systematic review-based approach. Ann Oncol (2019) 30(8):1232–43. 10.1093/annonc/mdz116 31056702

[28] Gibbons DL Creighton CJ Pan-cancer survey of epithelial-mesenchymal transition markers across the Cancer Genome Atlas. Dev Dyn (2018) 247:555–64. 10.1002/dvdy.24485 PMC5503821 28073171

[29] Lu YC Robbins PF Targeting neoantigens for cancer immunotherapy. Int Immunol (2016) 28:365–70. 10.1093/intimm/dxw026 PMC5007611 27208041

[30] Wilkinson NW Yothers G Lopa S Costantino JP Petrelli NJ Wolmark N Long-term survival results of surgery alone versus surgery plus 5-fluorouracil and leucovorin for stage II and stage III colon cancer: pooled analysis of NSABP C-01 through C-05. A baseline from which to compare modern adjuvant trials. Ann Surg Oncol (2010) 17(4):959–66. 10.1245/s10434-009-0881-y PMC2935319 20082144

[31] Zhou R Zeng D Zhang J Sun H Wu J Li N A robust panel based on tumour microenvironment genes for prognostic prediction and tailoring therapies in stage I-III colon cancer. EBioMedicine (2019) 42:420–30. 10.1016/j.ebiom.2019.03.043 PMC6491960 30917936

[32] Wang BD Lee NH Aberrant RNA Splicing in Cancer and Drug Resistance. Cancers (Basel) (2018) 10(11):458. 10.3390/cancers10110458 PMC6266310 30463359

[33] Ganesh K Stadler ZK Cercek A Mendelsohn RB Shia J Segal NH Immunotherapy in colorectal cancer: rationale, challenges and potential. Nat Rev Gastroenterol Hepatol (2019) 16(6):361–75. 10.1038/s41575-019-0126-x PMC7295073 30886395

[34] Suman S Das TP Sirimulla S Alatassi H Ankem MK Damodaran C Withaferin-A suppress AKT induced tumor growth in colorectal cancer cells. Oncotarget (2016) 7(12):13854–64. 10.18632/oncotarget.7351 PMC4924683 26883103

[35] Gurzu S Silveanu C Fetyko A Butiurca V Kovacs Z Jung I Systematic review of the old and new concepts in the epithelial-mesenchymal transition of colorectal cancer. World J Gastroenterol (2016) 22(30):6764–75. 10.3748/wjg.v22.i30.6764 PMC4974578 27570416

[36] Vodenkova S Buchler T Cervena K Veskrnova V Vodicka P Vymetalkova V 5-fluorouracil and other fluoropyrimidines in colorectal cancer: Past, present and future. Pharmacol Ther (2019) 206:107447. 10.1016/j.pharmthera.2019.107447 31756363

[37] Zhao X Fan J Zhi F Li A Li C Berger AE Mobilization of epithelial mesenchymal transition genes distinguishes active from inactive lesional tissue in patients with ulcerative colitis. Hum Mol Genet (2015) 24(16):4615–24. 10.1093/hmg/ddv192 26034135

[38] Pavlova NN Thompson CB The Emerging Hallmarks of Cancer Metabolism. Cell Metab (2016) 23:27–47. 10.1016/j.cmet.2015.12.006 26771115 PMC4715268

[39] Sharma A Boise LH Shanmugam M Cancer Metabolism and the Evasion of Apoptotic Cell Death. Cancers (Basel) (2019) 11(8):1144. 10.3390/cancers11081144 PMC6721599 31405035

[40] Guo W Tan HY Chen F Wang N Feng Y Targeting Cancer Metabolism to Resensitize Chemotherapy: Potential Development of Cancer Chemosensitizers from Traditional Chinese Medicines. Cancers (Basel) (2020) 12(2):404. 10.3390/cancers12020404 PMC7072159 32050640

[41] Gong W Ekmu B Wang X Lu Y Wan L AGR2-induced glucose metabolism facilitated the progression of endometrial carcinoma via enhancing the MUC1/HIF-1alpha pathway. Hum Cell (2020) 33(3):790–800. 10.1007/s13577-020-00356-4 32304027

[42] Trehoux S Duchene B Jonckheere N Van Seuningen I The MUC1 oncomucin regulates pancreatic cancer cell biological properties and chemoresistance. Implication of p42-44 MAPK, Akt, Bcl-2 and MMP13 pathways. Biochem Biophys Res Commun (2015) 456(3):757–62. 10.1016/j.bbrc.2014.12.025 25511698

[43] Yoshida GJ Metabolic reprogramming: the emerging concept and associated therapeutic strategies. J Exp Clin Cancer Res (2015) 34:111. 10.1186/s13046-015-0221-y 26445347 PMC4595070

[44] Mo X Huang X Feng Y Wei C Liu H Ru H Immune infiltration and immune gene signature predict the response to fluoropyrimidine-based chemotherapy in colorectal cancer patients. Oncoimmunology (2020) 9(1):1832347. 10.1080/2162402X.2020.1832347 33117604 PMC7575007

[45] Benatti P Gafa R Barana D Marino M Scarselli A Pedroni M Microsatellite instability and colorectal cancer prognosis. Clin Cancer Res (2005) 11(23):8332–40. 10.1158/1078-0432.CCR-05-1030 16322293

[46] Kamps R Brandão RD Bosch BJ Paulussen AD Xanthoulea S Blok MJ Next-Generation Sequencing in Oncology: Genetic Diagnosis, Risk Prediction and Cancer Classification. Int J Mol Sci (2017) 18(2):308. 10.3390/ijms18020308 PMC5343844 28146134

[47] Di Resta C Ferrari M Next Generation Sequencing: From Research Area to Clinical Practice. Ejifcc (2018) 29:215–20. PMC6247137 30479607

[48] Kuo FC Mar BG Lindsley RC Lindeman NI The relative utilities of genome-wide, gene panel, and individual gene sequencing in clinical practice. Blood (2017) 130(4):433–9. 10.1182/blood-2017-03-734533 PMC5813726 28600338

