# Supplementary figures and images for: A Combined Epithelial Mesenchymal Transformation and DNA Repair Gene Panel in Colorectal Cancer With Prognostic and Therapeutic Implication
Source: Front Oncol. 2021 Jan 15;10:595182. doi: 10.3389/fonc.2020.595182 (PMC7843609; doi:10.3389/fonc.2020.595182)

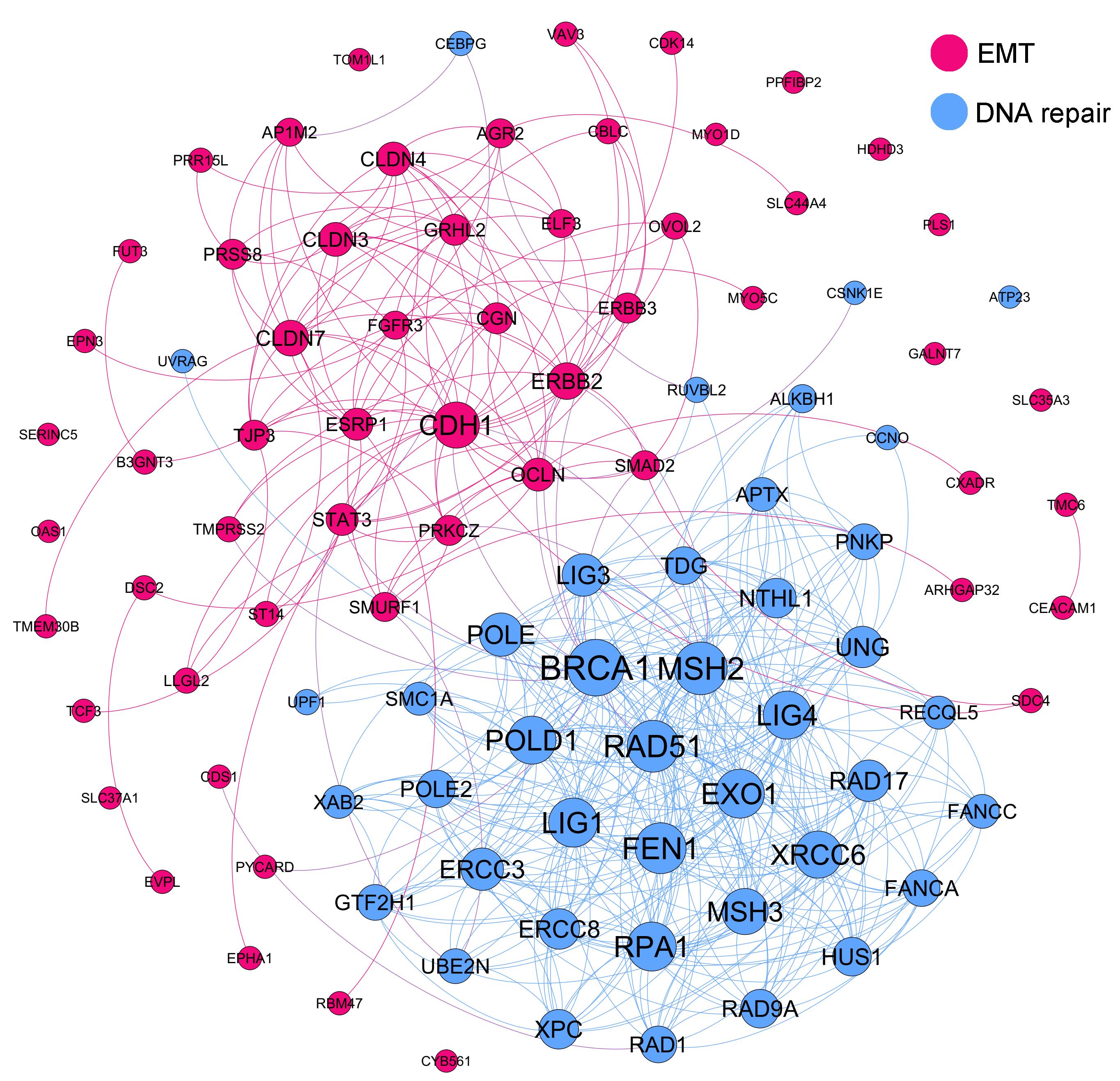

Supplement: Supplementary Figure 1 — Protein-protein interaction (PPI) network of the 98 EMT and DNA repair genes. Genes belonging to EMT are represented in red and Genes belonging to DNA repair are represented in blue. The size of a gene is positively correlated with the number of genes it links. [file Image_1.tif]

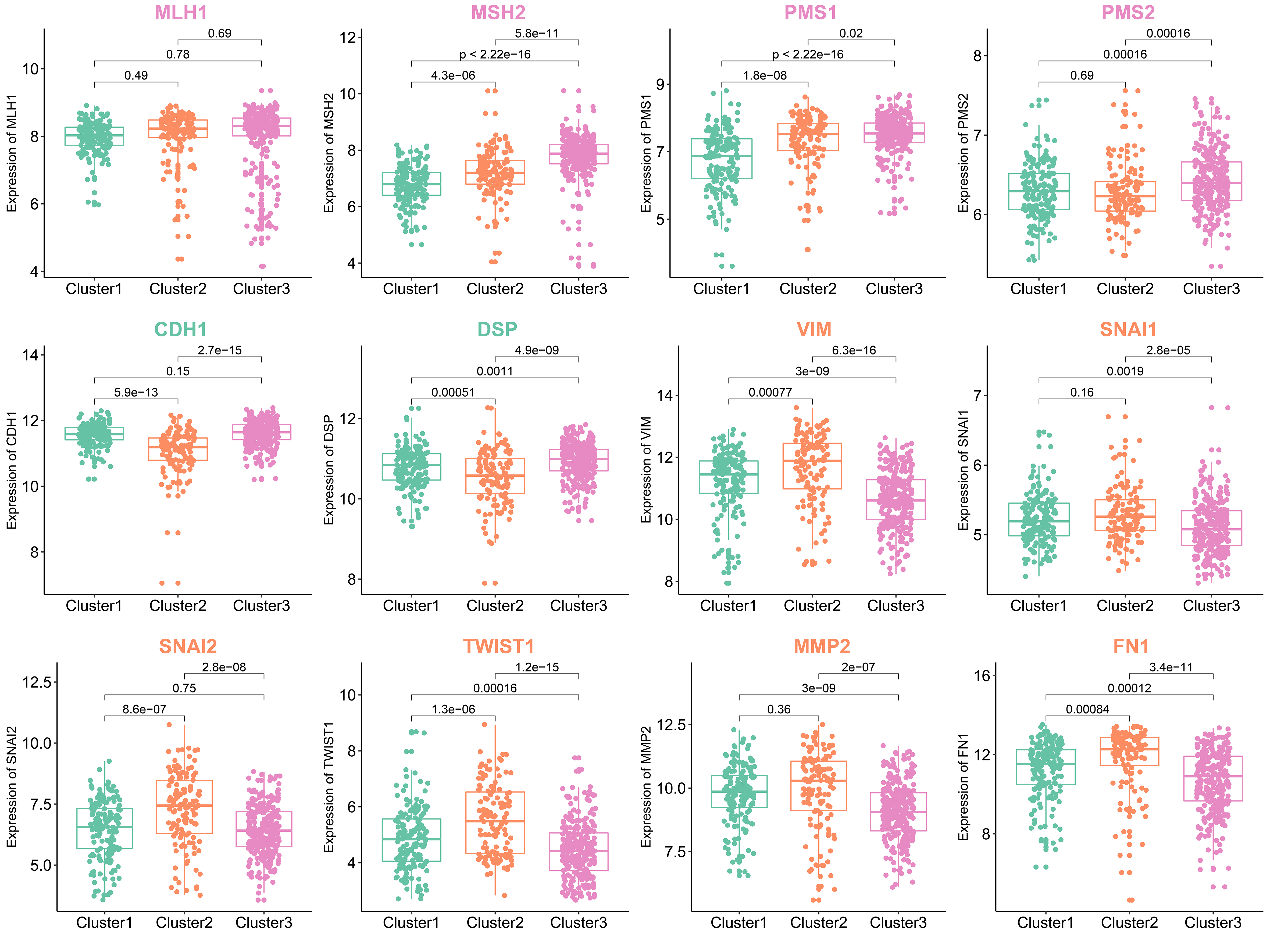

Supplement: Supplementary Figure 2 — Expression of representative EMT and DNA repair genes in the three CRC clusters. DNA repair genes were marked red and epithelial genes were green. Mesenchymal genes were orange. [file Image_2.tif]

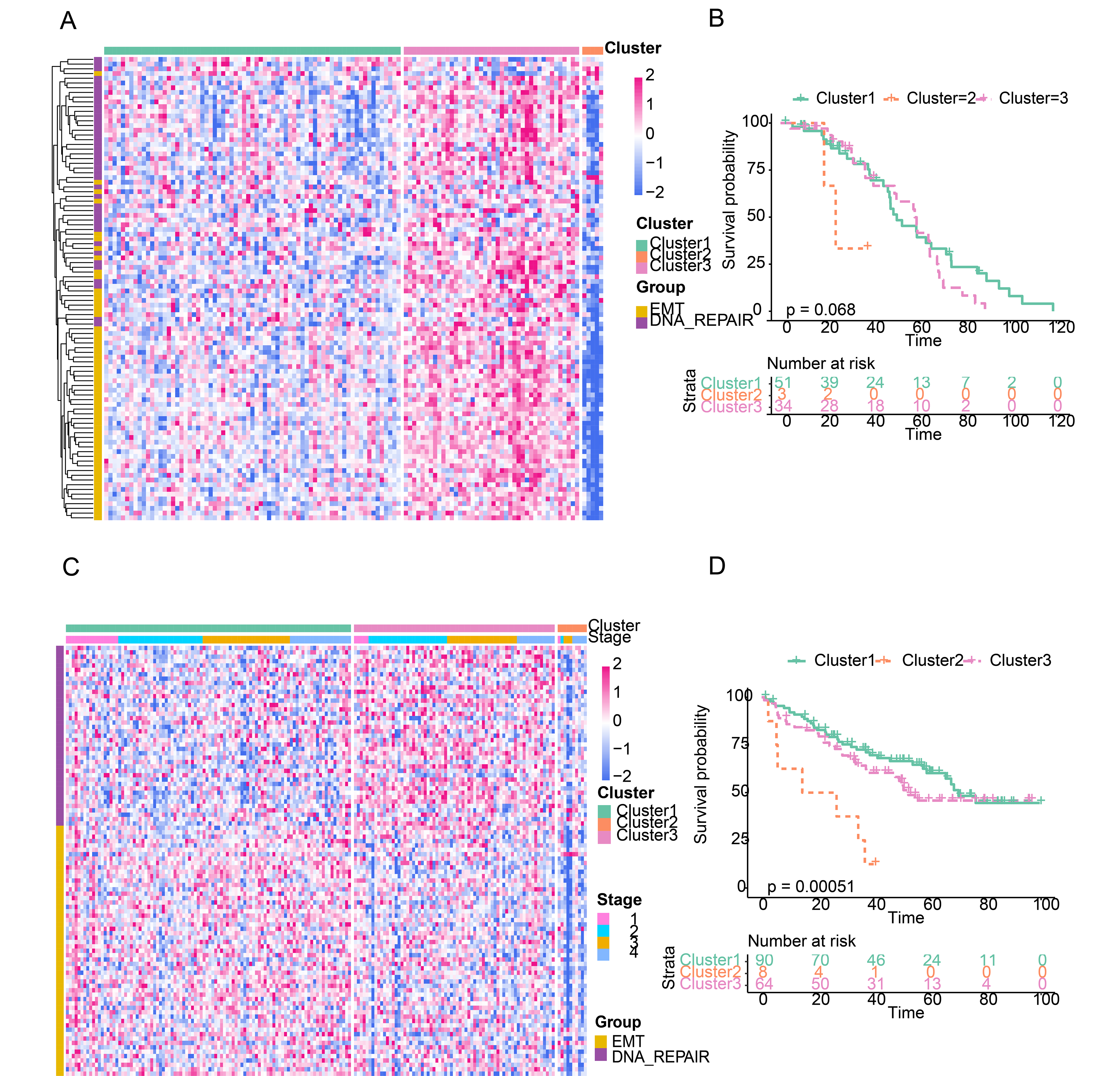

Supplement: Supplementary Figure 3 — Identification of distinct molecular clusters based on EMT and DNA repair genes using GSE14333 and GSE17536. (A) Heatmaps show the expression of 98 EMT and DNA repair genes (GSE14333). (B) Survival analysis of CRC patients in the three clusters (GSE14333). (C) Heatmaps show the expression of 98 EMT and DNA repair genes (GSE17536). D: Survival analysis of CRC patients in the three clusters (GSE17536). [file Image_3.tif]

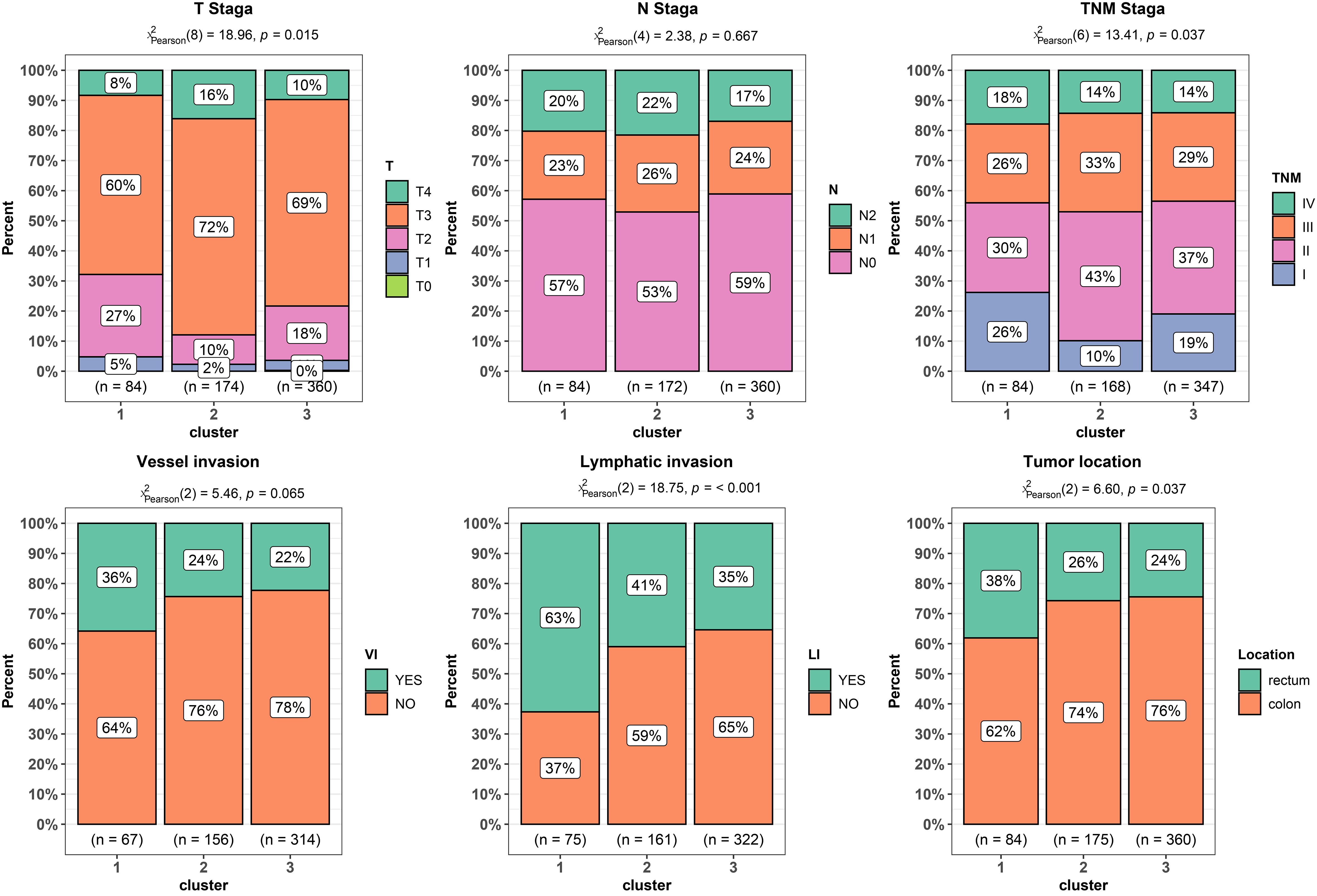

Supplement: Supplementary Figure 4 — Clinical characteristics of colorectal cancer (CRC) patients according to the cluster (TCGA data). Bar plots showing the proportion of tumor stage, tumor localization, lymphatic invasion (LV) and vessel invasion (VL) in different clusters. [file Image_4.tif]

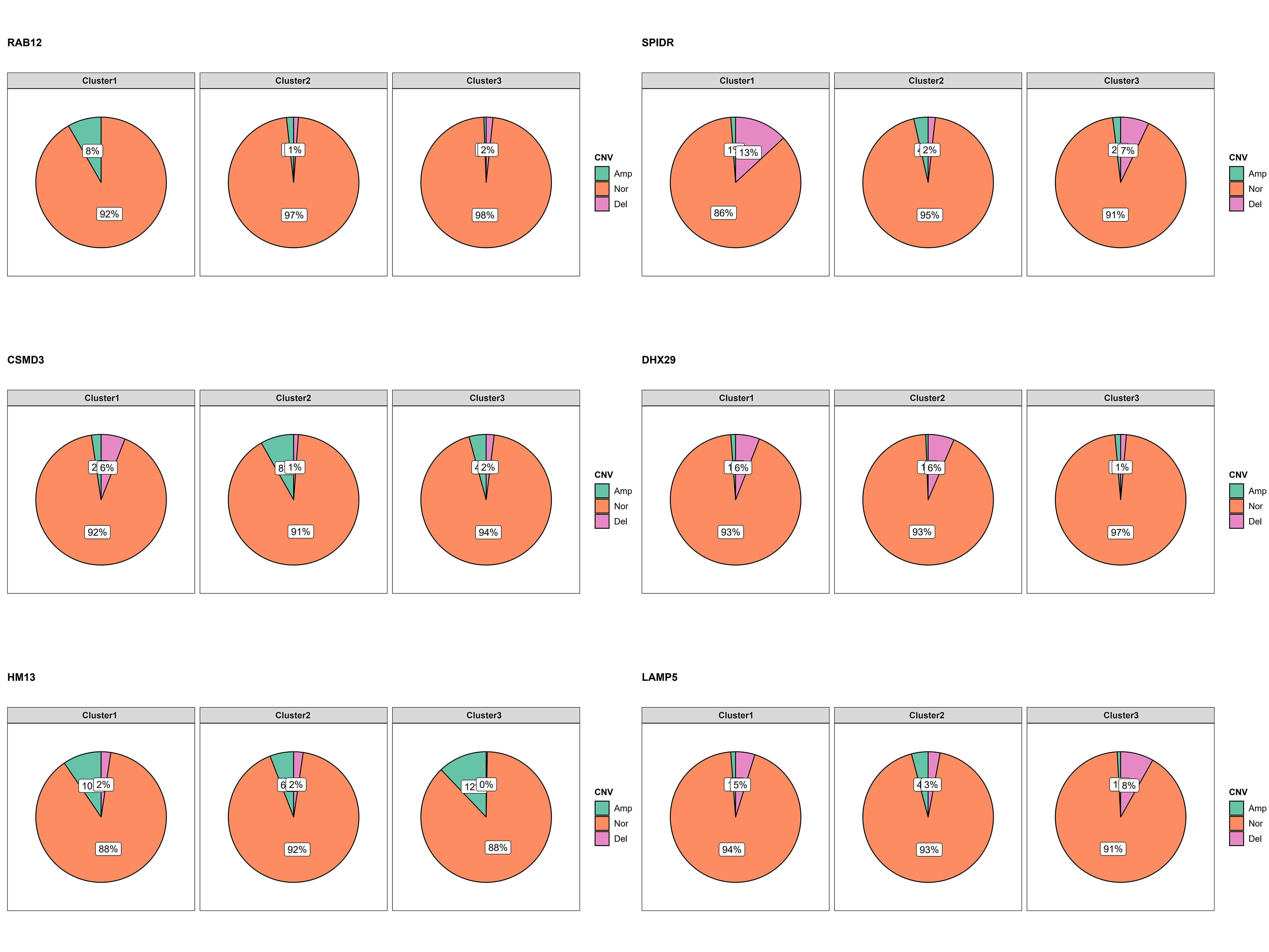

Supplement: Supplementary Figure 5 — Representative genes with significant differences in amplification or deletion frequency among the three clusters. [file Image_5.tif]

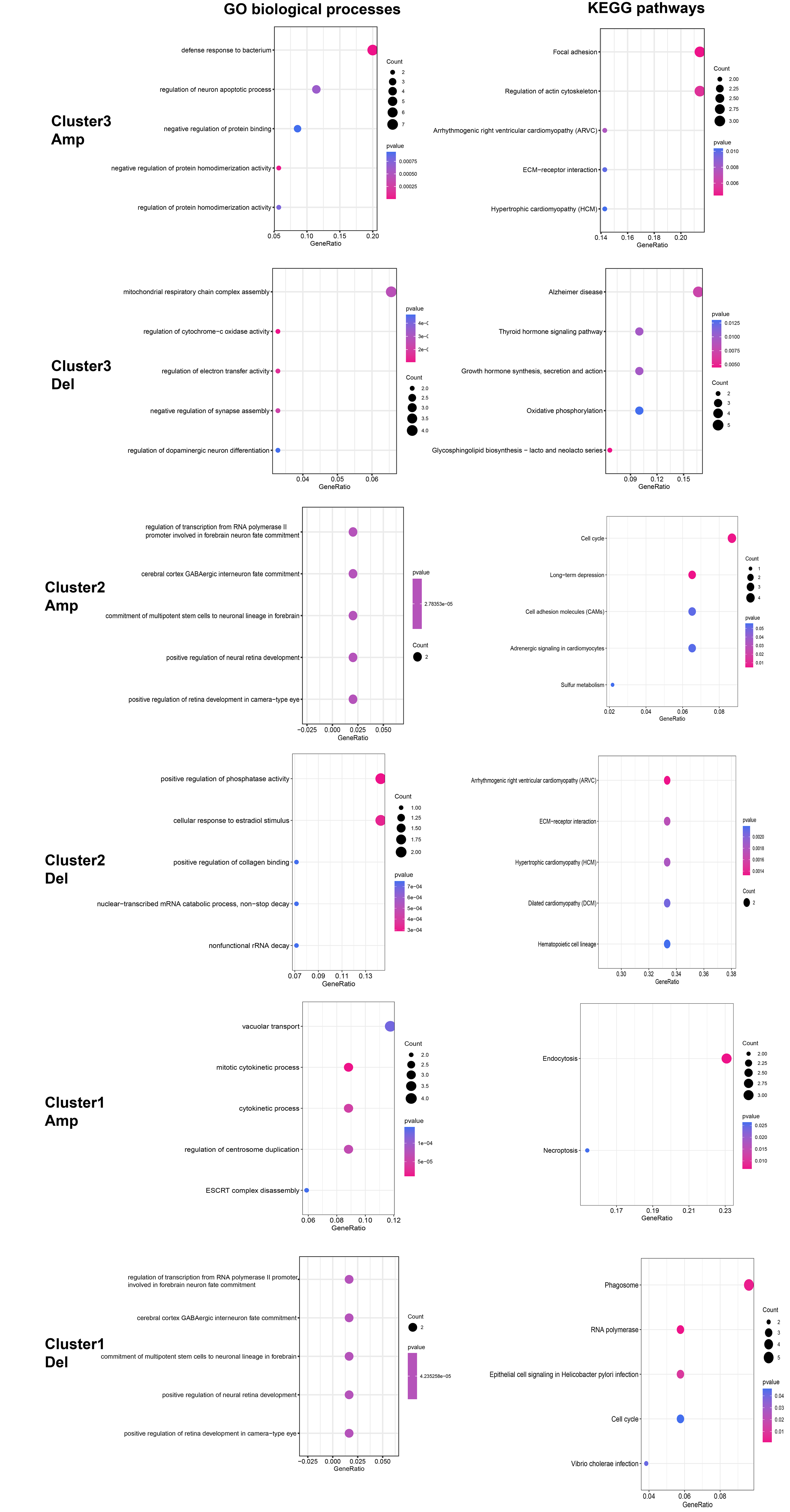

Supplement: Supplementary Figure 6 — Enrichment analysis of aberrant amplification or deletion of genes. Showing the top five terms with a P value less than 0.05. Left panel: GO biological process; Right panel: KEGG pathways. [file Image_6.tif]

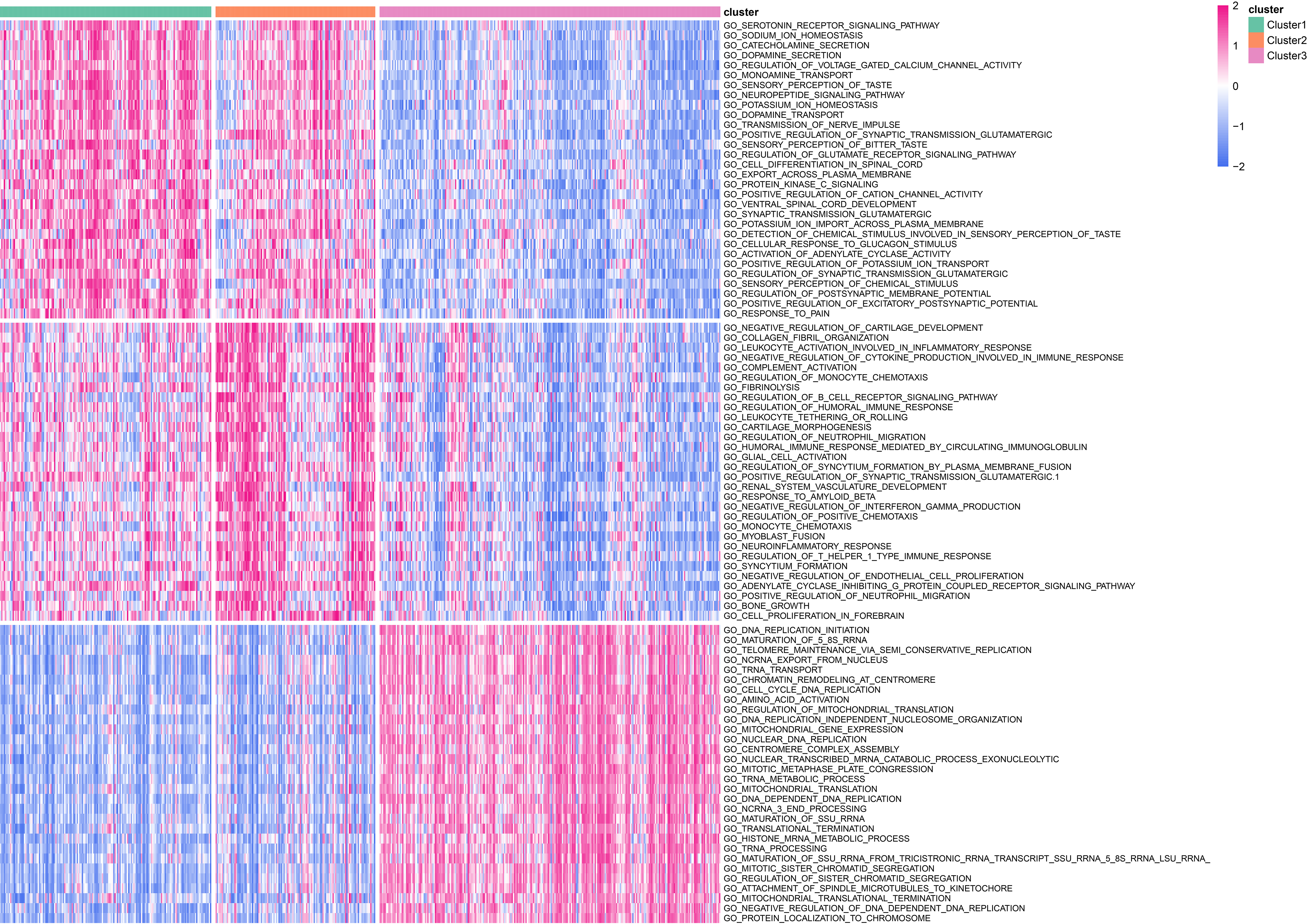

Supplement: Supplementary Figure 7 — Heatmap of different biological processes among the three clusters. Each cluster exhibit 30 of the most distinctive GO biological processes. [file Image_7.tif]

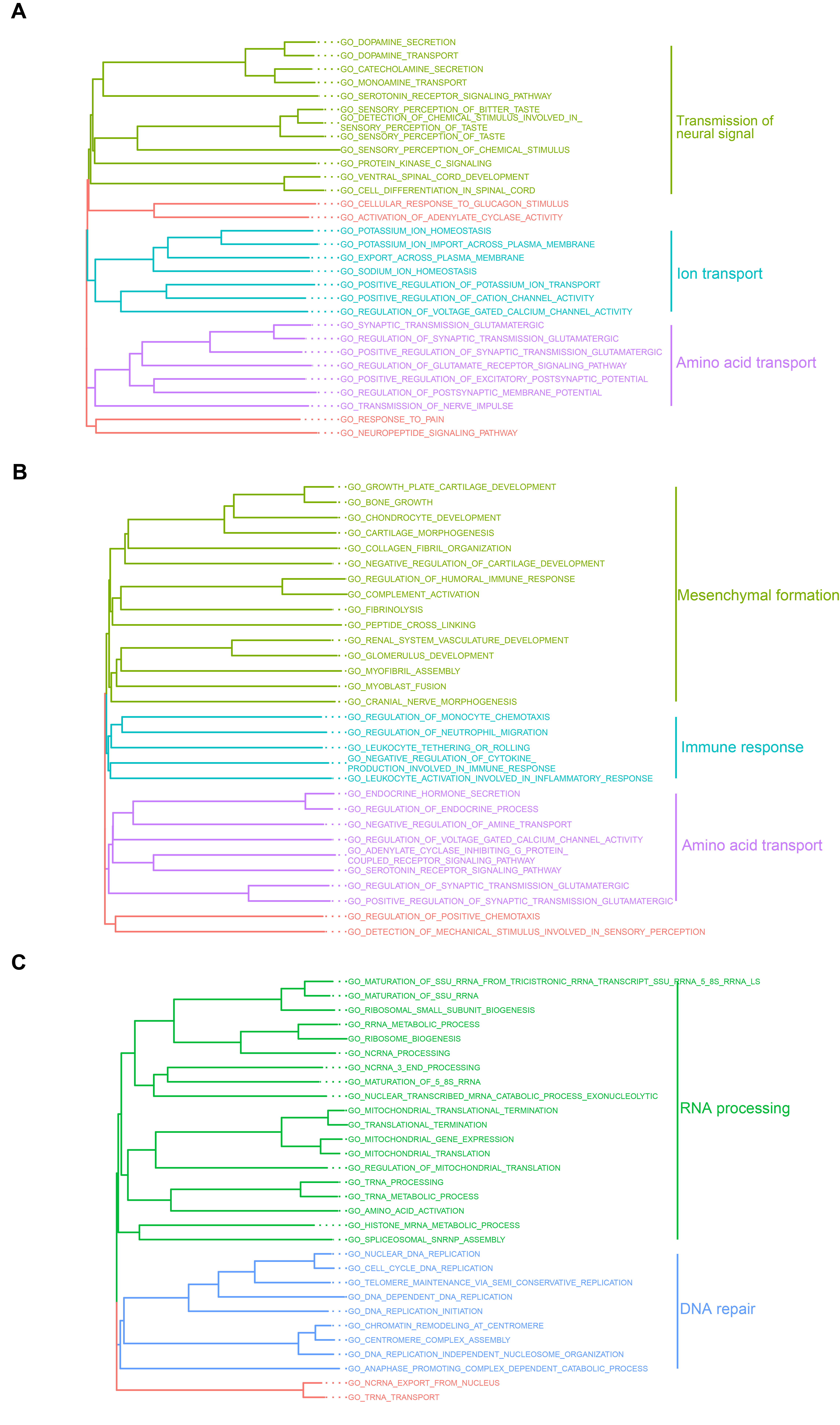

Supplement: Supplementary Figure 8 — Tree diagram of distinctive GO biological processes in each cluster. Clustering of GO terms according to the common genes contained in the different terms. The closer the two terms are, the more genes they share. (A) Cluster1; (B) Cluster2; (C) Cluster3. [file Image_8.tif]

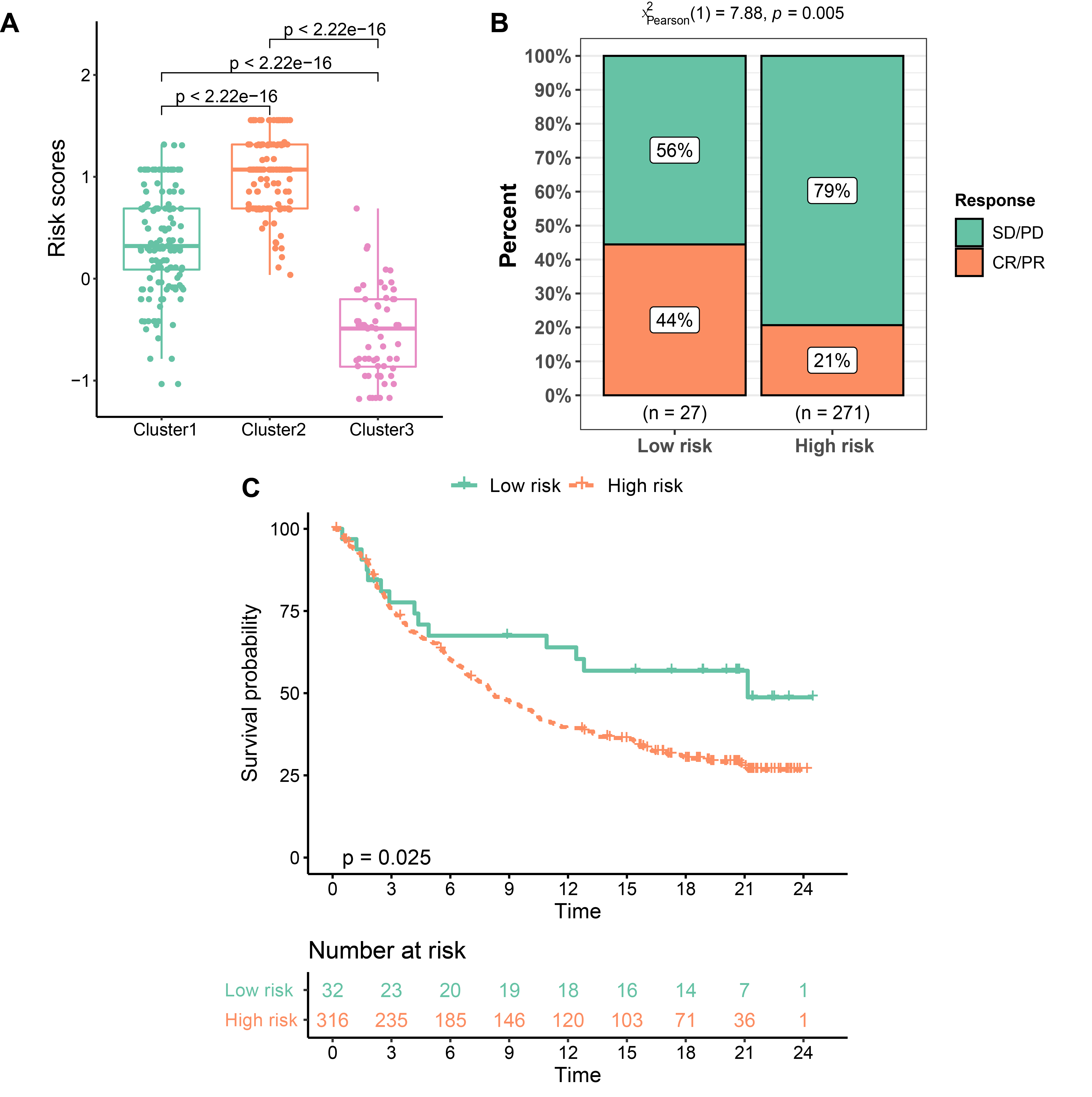

Supplement: Supplementary Figure 10 — Correlation between risk score and immunotherapy response. (A) Comparison of risk scores across clusters based on “IMvigor” dataset. (B) Response rate of patients to immunotherapy. Patients were stratified according the risk scores. CR, complete response; PR, partial response; SD, stable disease; PD, progressive disease. (C) Kaplan–Meier curves for patients receiving immunotherapy. [file Image_10.tif]
