# Supplementary material for: A Combined Epithelial Mesenchymal Transformation and DNA Repair Gene Panel in Colorectal Cancer With Prognostic and Therapeutic Implication
Source: Front Oncol. 2021 Jan 15;10:595182. doi: 10.3389/fonc.2020.595182 (PMC7843609; doi:10.3389/fonc.2020.595182)
Supplement: Supplementary file 12 [file Table_2.docx]

Supplementary Table 2: The relationships between CRC classifications and clinical characteristics in the GSE39582*.

| Feature | Cluster1 (N=168) | Cluster2  N= (127) | Cluster3  N= (271) | P-values |
| --- | --- | --- | --- | --- |
| Age (mean (SD)) | 66.73(12.48) | 64.72(12.34) | 68.03(14.05) | 0.067 |
| Sex (%) |  |  |  | 0.454 |
| Male | 93(55.4) | 75(59.1) | 142(52.4) |  |
| Female | 75(44.6) | 52(40.9) | 129(47.6) |  |
| BRAF mutation (%) |  |  |  | 0.022 |
| M | 6(3.6) | 14(11.0) | 31(11.4) |  |
| WT | 138(82.1) | 94(74.0) | 229(84.5) |  |
| NA | 24(14.3) | 19(15.0) | 11(4.1) |  |
| Chemotherapy type (%) |  |  |  | 0.129 |
| 5FU | 28(16.7) | 19(15.0) | 32(11.8) |  |
| FOLFIRI | 4(2.4) | 4(3.1) | 3(1.1) |  |
| FOLFOX | 3(1.8) | 9(7.1) | 11(4.1) |  |
| FUFOL | 20(11.9) | 6(4.7) | 25(9.2) |  |
| other | 0(0.0) | 1(0.8) | 2(0.7) |  |
| NA | 113(67.3) | 88(69.3) | 198(73.1) |  |
| CIMP status (%) |  |  |  | 0.003 |
| - | 127(75.6) | 78(61.4) | 200(73.8) |  |
| + | 13(7.7) | 26(20.5) | 52(19.2) |  |
| NA | 28(16.7) | 23(18.1) | 19(7.0) |  |
| CIN status (%) |  |  |  | <0.001 |
| - | 12(7.1) | 27(21.3) | 71(26.2) |  |
| + | 113(67.3) | 66(52.0) | 175(64.6) |  |
| NA | 43(25.6) | 34(26.8) | 25(9.2) |  |
| CIT subtype (%) |  |  |  | <0.001 |
| C1 | 42(25.0) | 12(9.4) | 62(22.9) |  |
| C2 | 12(7.1) | 25(19.7) | 67(24.7) |  |
| C3 | 20(11.9) | 8(6.3) | 47(17.3) |  |
| C4 | 8(4.8) | 46(36.2) | 5(1.8) |  |
| C5 | 49(29.2) | 29(22.8) | 74(27.3) |  |
| C6 | 37(22.0) | 7(5.5) | 16(5.9) |  |
| KRAS mutation (%) |  |  |  | 0.987 |
| M | 63(37.5) | 50(39.4) | 104(38.4) |  |
| WT | 97(57.7) | 74(58.3) | 157(57.9) |  |
| NA | 8(4.8) | 3(2.4) | 10(3.7) |  |
| MMR status (%) |  |  |  | <0.001 |
| dMMR | 3(1.8) | 15(11.8) | 57(21.0) |  |
| pMMR | 152(90.5) | 104(81.9) | 188(69.4) |  |
| NA | 13(7.7) | 8(6.3) | 26(9.6) |  |
| T stage (%) |  |  |  | <0.001 |
| T0 | 0(0.0) | 0(0.0) | 1(0.4) |  |
| T1 | 0(0.0) | 6(4.7) | 5(1.8) |  |
| T2 | 4(2.4) | 7(5.5) | 34(12.5) |  |
| T3 | 113(67.3) | 71(55.9) | 183(67.5) |  |
| T4 | 39(23.2) | 34(26.8) | 46(17.0) |  |
| Tis | 0(0.0) | 2(1.6) | 1(0.4) |  |
| NA | 12(7.1) | 7(5.5) | 1(0.4) |  |
| N stage (%) |  |  |  | <0.001 |
| N+ | 2(1.2) | 3(2.4) | 1(0.4) |  |
| N0 | 71(42.3) | 60(47.2) | 171(63.1) |  |
| N1 | 46(27.4) | 22(17.3) | 66(24.4) |  |
| N2 | 35(20.8) | 32(25.2) | 31(11.4) |  |
| N3 | 2(1.2) | 3(2.4) | 1(0.4) |  |
| NA | 12(7.1) | 7(5.5) | 1(0.4) |  |
| M stage (%) |  |  |  | <0.001 |
| M0 | 141(83.9) | 91(71.7) | 250(92.3) |  |
| M1 | 15(8.9) | 28(22.0) | 18(6.6) |  |
| MX | 0(0.0) | 1(0.8) | 2(0.7) |  |
| NA | 12(7.1) | 7(5.5) | 1(0.4) |  |
| TNM stage (%) |  |  |  | <0.001 |
| 0 | 0(0.0) | 2(1.6) | 2(0.7) |  |
| I | 0(0.0) | 8(6.3) | 25(9.2) |  |
| II | 77(45.8) | 47(37.0) | 140(51.7) |  |
| III | 77(45.8) | 42(33.1) | 86(31.7) |  |
| IV | 14(8.3) | 28(22.0) | 18(6.6) |  |
| TNM stage (%) |  |  |  | 0.001 |
| 0-II | 77(45.8) | 57(44.9) | 167(61.6) |  |
| III_IV | 91(54.2) | 70(55.1) | 104(38.4) |  |
| TP53 mutation (%) |  |  |  | 0.392 |
| M | 66(39.3) | 33(26.0) | 91(33.6) |  |
| WT | 45(26.8) | 30(23.6) | 86(31.7) |  |
| NA | 57(33.9) | 64(50.4) | 94(34.7) |  |
| Tumor location (%) |  |  |  | 0.024 |
| Proximal | 52(31.0) | 54(42.5) | 118(43.5) |  |
| Distal | 116(69.0) | 73(57.5) | 153(56.5) |  |

*: Missing data is not included in the statistical test.

NA: missing data; CIMP: CpG island methylator phenotype; CIN: Chromosomal instability; dMMR: deficient mismatch repair; pMMR: Mismatch Repair Proficient; CIT: Cartes d’Identite´ des Tumeurs program;
